# Comparing TAVR + PCI vs. SAVR + CABG across short- and mid- to long-term horizons in patients with severe aortic stenosis and concomitant CAD: a systematic review and meta-analysis

**DOI:** 10.3389/fcvm.2026.1640906

**Published:** 2026-01-30

**Authors:** Xiang Fang, Xuge Zhang, Fei Wei, Shenghong Feng, Xiaomei Chen

**Affiliations:** 1 Department of Cardiology, Dazhou Second People’s Hospital, Dazhou, Sichuan, China; 2 Department of Otorhinolaryngology Head and Neck Surgery, Dazhou Second People’s Hospital, Dazhou, Sichuan, China

**Keywords:** aortic stenosis, coronary artery bypass grafting, coronary artery disease, meta-analysis, percutaneous coronary intervention, surgical aortic valve replacement, transcatheter aortic valve replacement

## Abstract

**Background:**

The optimal revascularization approach for intermediate- and high-risk individuals with severe aortic stenosis (AS) and concomitant coronary artery disease (CAD) remains uncertain, particularly regarding the comparative short- and mid- to long-term outcomes of transcatheter aortic valve replacement with percutaneous coronary intervention (TAVR + PCI) vs. surgical aortic valve replacement with coronary artery bypass grafting (SAVR + CABG).

**Methods:**

A systematic search of major databases was conducted up to March 2025 to identify studies comparing TAVR + PCI vs. SAVR + CABG in this population. Meta-analyses were performed using a random-effects model to estimate pooled odds ratios (ORs) and 95% confidence intervals (CIs). Evidence quality was assessed using the GRADE framework.

**Results:**

Thirteen studies comprising 53,869 patients were analyzed. Compared with SAVR + CABG, TAVR + PCI was associated with lower 30-day risks of stroke, myocardial infarction, and acute kidney injury, but higher permanent pacemaker implantation. No differences were found in all-cause mortality, major vascular complications, or major bleeding. In mid- to long-term follow-up (≥2 years), the TAVR + PCI group exhibited increased risks of all-cause mortality, myocardial infarction, and repeat revascularization, with similar stroke rates between strategies. Certainty of evidence ranged from very low to moderate.

**Conclusions:**

In intermediate- and high-risk patients with severe AS and concomitant CAD, TAVR + PCI appears to confer short-term safety advantages but may be associated with less favorable mid- to long-term outcomes compared with SAVR + CABG. These findings support individualized revascularization strategies that balance early procedural safety against longer-term risks and highlight the need for further randomized trials with extended follow-up.

**Systematic Review Registration:**

https://www.crd.york.ac.uk/PROSPERO/view/CRD420251000317, PROSPERO CRD420251000317.

## 1 Introduction

Severe aortic stenosis (AS) represents a prevalent valvular heart disease in the elderly, with incidence increasing significantly with age (1). Studies have reported that 15% to 81% of individuals with AS have concomitant coronary artery disease (CAD), with a higher prevalence observed in older populations (2–4). These patients experience increased cardiovascular risk due to impaired myocardial perfusion and left ventricular dysfunction (5, 6). Determining the optimal coronary revascularization approach in individuals undergoing aortic valve replacement remains a complex and unresolved clinical issue, particularly in those with advanced age and extensive coronary disease.

Traditionally, surgical aortic valve replacement (SAVR) with coronary artery bypass grafting (CABG) has been considered the standard approach for eligible individuals, as it provides complete revascularization and durable valve function. Accordingly, SAVR combined with CABG has historically been favored in younger patients and those with acceptable surgical risk profiles (5, 6). However, SAVR + CABG is associated with higher surgical trauma, prolonged recovery, and increased perioperative complications, particularly in intermediate- and high-risk individuals. Recent advances in transcatheter aortic valve replacement (TAVR) and percutaneous coronary intervention (PCI) have facilitated the widespread adoption of TAVR + PCI as a minimally invasive alternative, especially in elderly and high-risk populations (3, 7). According to the 2025 ESC/EACTS Guidelines for the Management of Valvular Heart Disease, TAVR is recommended for anatomically suitable patients aged ≥70 years with tricuspid severe AS, while the choice between TAVR and SAVR in other candidates should be guided by a multidisciplinary Heart Team integrating clinical risk, anatomical feasibility, imaging findings, and patient preference (6). These evolving treatment strategies underscore the need for comparative evidence on the clinical outcomes of TAVR + PCI vs. SAVR + CABG in individuals with AS and concomitant CAD (8, 9).

Several studies have evaluated TAVR + PCI, with some comparing it to SAVR + CABG (10–13). However, most evidence has focused on short-term outcomes (e.g., mortality, stroke, acute kidney injury), while evidence beyond the early post-procedural period remains limited and heterogeneous (14–17). Although some studies have reported outcomes beyond two years, their findings are highly heterogeneous. From a clinical perspective, outcomes beyond two years are more appropriately considered mid-term rather than definitive long-term endpoints, particularly in the context of valve durability and coronary disease progression (17). Additionally, substantial heterogeneity exists across studies regarding patient risk stratification, revascularization strategies, and study designs, contributing to variability in reported outcomes (14). In particular, for patients with complex CAD (e.g., high SYNTAX scores), PCI may result in incomplete revascularization, potentially compromising mid- to long-term prognosis, whereas CABG may provide more sustained hemodynamic benefits (18). Against this background of heterogeneous and incomplete primary evidence, a rigorous synthesis of available data remains essential to clarify the relative benefits and limitations of contemporary interventional and surgical strategies.

Several meta-analyses have previously synthesized evidence comparing TAVR + PCI with SAVR + CABG in patients with AS and concomitant CAD, including those by Sakurai et al. (14), Baudo et al. (19), and Emara et al. (20). While these studies provided important insights into perioperative outcomes and overall survival, they differed substantially in inclusion periods, surgical risk profiles, follow-up definitions, and analytical approaches. In particular, most prior syntheses pooled heterogeneous patient populations across a broad risk spectrum or emphasized aggregate outcomes without predefined temporal stratification, thereby limiting their applicability to contemporary, guideline-based decision-making in intermediate- and high-risk patients. In contrast, the present study focuses specifically on intermediate- and high-risk individuals, as operationally defined by contemporary surgical risk models and clinical characteristics, and applies a structured comparison across predefined short-term and ≥2-year follow-up horizons. By incorporating evidence exclusively from randomized controlled trials and high-quality cohort studies, this meta-analysis aims to reduce clinical heterogeneity, enhance temporal interpretability of outcomes, and align evidence synthesis with current clinical practice patterns and evolving guideline recommendations.

This meta-analysis aims to systematically compare short- and mid- to long-term outcomes of TAVR + PCI vs. SAVR + CABG in intermediate- and high-risk patients with severe AS and concomitant CAD. By incorporating evidence from randomized controlled trials (RCTs) and cohort studies, we seek to address existing heterogeneity and generate clinically relevant, methodologically rigorous conclusions to inform treatment selection, guide personalized decision-making, and support future updates to practice guidelines.

## 2 Methods

### 2.1 Protocol registration

This meta-analysis was conducted in adherence to the Cochrane Handbook and PRISMA 2020 guidelines (21), and was prospectively registered in PROSPERO (CRD420251000317). The PRISMA 2020 checklist is provided in Supplementary Table S1.

### 2.2 Search strategy

A comprehensive literature search was performed in PubMed, Embase, Web of Science, and the Cochrane Library up to March 1, 2025. The search was guided by the PICOS framework, encompassing Population, Intervention, Comparison, Outcomes, and Study design. Relevant MeSH terms and free-text keywords related to the interventions and comparators—such as “aortic stenosis”, “TAVR”, “PCI”, “SAVR”, and “CABG”—were employed. No restrictions were imposed on population, outcome, or study design to ensure comprehensive coverage. Supplementary Table S2 outlines the complete search strategy applied across all databases.

### 2.3 Inclusion and exclusion criteria

Study eligibility was determined according to predefined PICOS criteria:

(i) **Population**

Severe AS: defined by at least one of the following: Imaging (e.g., echocardiography, computed tomography, magnetic resonance imaging) demonstrating an aortic valve area < 1.0 cm^2^ or mean pressure gradient ≥ 40 mmHg; confirmed by catheter-based or surgical intervention as severe aortic stenosis.

Coexisting CAD: Presence of at least one diameter stenosis > 70% in major coronary arteries and/or >50% diameter stenosis in the left main coronary artery, as assessed by invasive coronary angiography. Coronary computed tomography angiography findings were considered only if confirmed by invasive angiography or functionally significant (fractional flow reserve ≤ 0.80 or instantaneous wave-free ratio ≤ 0.89).

Risk Stratification: Intermediate- and high-risk individuals were identified based on surgical risk, clinical characteristics, and anatomical burden, meeting at least one of the following criteria:

(1) Age ≥75 years; (2) age ≥70 years with additional high-risk features (e.g., frailty, significant comorbidities); (3) EuroSCORE II ≥4% or STS score ≥4%; (4) multivessel or complex coronary artery disease (e.g., SYNTAX score >22, left main plus LAD disease, or three-vessel disease); (5) comorbidities known to increase surgical risk—such as diabetes, chronic kidney disease with eGFR <30 mL/min/1.73 m^2^, chronic obstructive pulmonary disease, malignancy, or cognitive impairment; (6) left ventricular ejection fraction ≤35%; (7) New York Heart Association class III or IV.

These criteria were used to operationalize intermediate- or high-risk status for study inclusion, informed by established surgical risk models (including STS-PROM and EuroSCORE II) and the conceptual framework of contemporary guidelines, rather than to define treatment-recommendation thresholds.

(ii) **Intervention**
  TAVR+PCI
(iii) **Comparison**
  SAVR+CABG
(iv) **Outcomes**

Short-term outcomes: 30-day all-cause mortality, 30-day myocardial infarction, 30-day stroke, 30-day acute kidney injury, 30-day major vascular complications, 30-day major bleeding, and 30-day permanent pacemaker implantation.

Mid- to long-term outcomes (≥2 years): all-cause mortality during follow-up, myocardial infarction during follow-up, revascularization during follow-up, stroke during follow-up.

Outcome definitions were prespecified to follow those reported in the original studies. To avoid potential bias, no *post hoc* reclassification was performed. To ensure transparency, all outcome definitions were systematically extracted and are summarized in Supplementary Table S3 (short-term outcomes) and Supplementary Table S4 (mid- to long-term outcomes), allowing readers to assess potential heterogeneity.

(v) **Study design**

Published RCTs and cohort studies.

**Exclusion Criteria**: Low-risk patients (EuroSCORE II <4% or STS score <4%) and studies assessing isolated TAVR, PCI, SAVR, or CABG were excluded. Hybrid revascularization strategies (e.g., minimally invasive or robotic-assisted CABG + PCI) were excluded. Studies without extractable data on short- or mid- to long-term outcomes were excluded. For overlapping patient populations, RCTs were prioritized over observational studies. If multiple observational studies shared overlapping populations, priority was given to those featuring a greater number of participants, extended duration of follow-up, or more rigorous statistical adjustments (e.g., propensity score matching) to minimize bias and ensure analytical consistency. Non-original research, including case reports, letters, editorials, conference abstracts, review articles, unpublished data, and non-English studies, was excluded.

### 2.4 Literature screening and data extraction

Data extraction was independently performed by XF and XGZ. Discrepancies were resolved through discussion or consultation with a third author (XMC) until consensus was reached. The extracted data included: author and year, country/region, study design, follow-up duration, sample size, patient age, sex, baseline comorbidities, TAVR approach, time interval between PCI and TAVR procedures, risk stratification, and clinical outcomes. The definitions of short-term and mid- to long-term outcomes were adopted as reported in the original studies. To enhance transparency and allow comparison of heterogeneity in event classification, the specific definitions used across included studies are summarized in Supplementary Table S3 (short-term outcomes) and Supplementary Table S4 (mid- to long-term outcomes).

### 2.5 Risk of bias within studies

For RCTs, the risk of bias was assessed using the Cochrane Collaboration's Risk of Bias tool, which evaluates five key domains: randomization process, allocation concealment, blinding, outcome data completeness, and reporting bias (22). For cohort studies, the Newcastle-Ottawa Scale (NOS) was employed to evaluate study quality across three domains: selection, comparability, and outcome assessment, with higher scores reflecting a lower risk of bias (23). Studies with scores ≥7 were considered of high quality. Discrepancies in the assessment process were resolved by consensus or, if consensus could not be reached, through arbitration by a third author.

### 2.6 Data analysis

Statistical analyses were performed using Review Manager (version 5.4, Cochrane Collaboration, Oxford, UK). For short-term binary outcomes, odds ratios (ORs) with 95% confidence intervals (CIs) were used. For mid- to long-term outcomes (≥2 years), hazard ratios (HRs) were prespecified as the preferred effect measure. Where HRs were not reported and Kaplan–Meier curves were unavailable for estimation, crude event counts were extracted and ORs were calculated as an alternative effect measure. Between-study heterogeneity was evaluated using Cochran's Q and the *I*^2^ statistic, with heterogeneity considered substantial if the *Q* test *P*-value was <0.10 or *I*^2^ exceeded 50%. In such cases, a random-effects model was applied to derive pooled estimates. Sensitivity analyses and risk of bias assessments were conducted to ensure result robustness. Where applicable, subgroup analyses explored sources of heterogeneity based on study design, follow-up time, and sample size. Funnel plots were generated in RevMan to visualize potential publication bias. For outcomes reported in ≥3 studies, Egger's regression test (Stata version 18.0, StataCorp) was used to quantify small-study effects, with *P* < 0.05 indicating significant asymmetry.

### 2.7 GRADE assessment

Evidence quality for both short- and mid- to long-term outcomes was appraised using the GRADE framework (Grading of Recommendations, Assessment, Development, and Evaluation), which considers study design, risk of bias, consistency, directness, precision, and publication bias. Based on these domains, evidence was rated as high, moderate, low, or very low quality (24).

## 3 Results

### 3.1 Study selection and Patients' characteristics

A comprehensive literature search identified 462 articles, including 67 from PubMed, 193 from Embase, 22 from the Cochrane Library, and 180 from Web of Science. After full-text review of 25 records, 10 conference abstracts and 2 studies defining short-term outcomes as those occurring during hospitalization were excluded, as they did not align with the 30-day post-operative definition used in this analysis. Ultimately, 13 studies (25–37) were included, comprising a total of 53,869 patients — 24,913 receiving TAVR + PCI and 28,956 undergoing SAVR + CABG.

**Figure 1:**
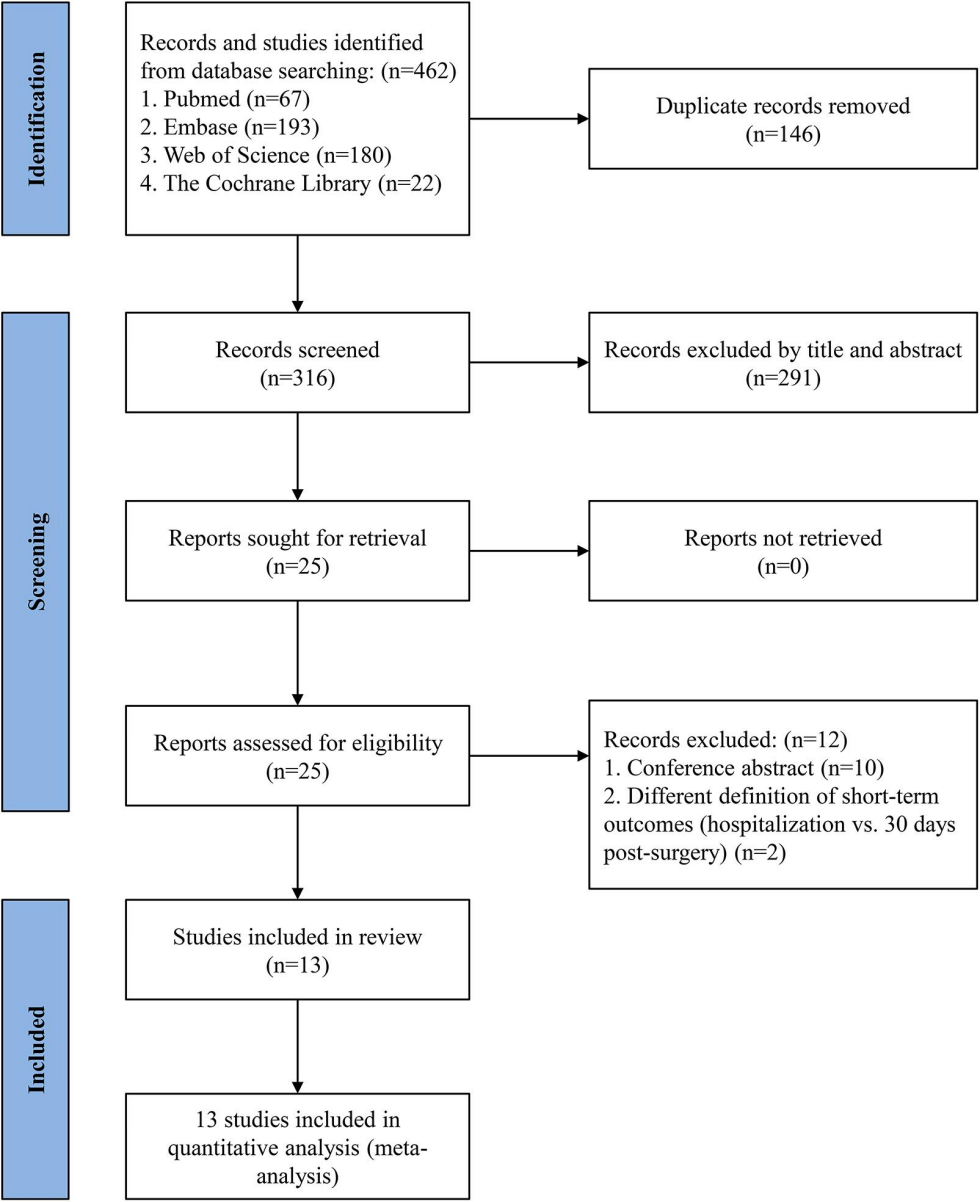
PRISMA flow diagram of study selection.

The 13 included studies comprised 2 RCTs (26, 34) and 11 cohort studies (25, 27–33, 35–37) (3 prospective and 8 retrospective). Included individuals had a mean age of 75.1 years, with 60.1% were being male (*n* = 32,377). The duration of follow-up varied across studies, ranging from 30 days to 9.2 years. A summary of study characteristics and patient demographics is presented in Tables 1, 2. Figure 1 outlines the study selection process, while the risk of bias assessment is detailed in Supplementary Figure S1 and Supplementary Table S5.

**Table 1:** Characteristics of included studies.

Author (Year)	Study design	Region	Intervention treatment	Control group	Total sample size (N)	TAVR approach	PCI-to-TAVR interval	Patient risk classification	Follow-up duration
Taghiyev et al. (25)	Retrospective cohort	Germany	TAVR + PCI	SAVR + CABG	80	Transfemoral	All PCI procedures were conducted within a 90-day window preceding TAVR or concurrently with the TAVR procedure	Intermediate risk	9.2 years
Kedhi et al. (26)	RCT	Europe	TAVR + FFR-guided PCI	SAVR + CABG	172	Transfemoral and transsubclavian	All PCI procedures were conducted within a 40-day window preceding TAVR or concurrently with the TAVR procedure	High risk	1 year
Jagadeesan et al. (27)	Retrospective cohort	United States	TAVR + PCI	SAVR + CABG	37,822	Transfemoral	Elective PCI performed within 3 months before or after TAVR; Non-emergent PCI conducted during TAVR hospitalization	Intermediate and high risk	5 years
Amat-Santos et al. (28)	Retrospective cohort	Spain	TAVR + PCI	SAVR + CABG	508	Transfemoral	All PCI procedures were conducted within a 40-day window preceding TAVR or concurrently with the TAVR procedure	Intermediate and high risk	5 years
Ullah et al. (29)	Retrospective cohort	United States	TAVR + PCI	SAVR + CABG	11,361	Transfemoral and transapical	All PCI procedures were performed either within a 3 months period preceding the TAVR procedure or concurrently with the TAVR procedure.	High risk	30 days
McInerney et al. (30)	Retrospective cohort	Spain	TAVR + PCI	SAVR + CABG	1,548	NA	All PCI procedures were performed within 6 months prior to TAVR	Intermediate and high risk	30 days
Lérault et al. (31)	Retrospective cohort	France	TAVR + PCI	SAVR + CABG	241	Transfemoral, transcarotid, transsubclavian, transaortic, and transapical	NA	High risk	3.3 years
Elderia et al. (32)	Retrospective cohort	Germany	TAVR + PCI	SAVR + CABG	478	Transfemoral, transapical, and transsubclavian/axillary	All PCI procedures were performed within 6 months prior to TAVR	High risk	30 days
Alperi et al. (33)	Retrospective cohort	North America and Europe	TAVR + PCI	SAVR + CABG	312	Transfemoral	All PCI procedures were performed within 3 months prior to TAVR or concurrently with the TAVR procedure	Intermediate risk	5 years
Søndergaard et al. (34)	RCT	United States, Canada, and Europe	TAVR + PCI	SAVR + CABG	332	Transfemoral and transapical	All PCI procedures were performed within 7 days prior to TAVR	Intermediate risk	2 years
Baumbach et al. (35)	Prospective cohort	Germany	TAVR + PCI	SAVR + CABG	576	Transfemoral and transapical	All PCI procedures were conducted within a 12 months window preceding TAVR	High risk	30 days
Barbanti et al. (36)	Prospective cohort	Italy	TAVR + PCI	SAVR + CABG	472	Transfemoral	All PCI procedures were performed within 6 months prior to TAVR, or PCI and TAVR performed concurrently in the same session	Intermediate and high risk	3 years
Wendt et al. (37)	Prospective cohort	Germany	TAVR + PCI	SAVR + CABG	243	Transfemoral and transapical	All PCI procedures were performed within 12 months before TAVR	High risk	6 years

TAVR, rranscatheter aortic valve replacement; PCI, percutaneous coronary intervention; SAVR, surgical aortic valve replacement; CABG, coronary artery bypass grafting; RCT, randomized controlled trial; FFR, fractional flow reserve; NA, not available.

**Table 2:** Patient demographics and baseline clinical characteristics.

Author (year)	Total sample size (*N*)	I/C (*n*)	Mean age (years) I/C	Male, *n* (%) I/C	Hypertension, *n* (%) I/C	Diabetes, *n* (%) I/C	CKD, *n* (%) I/C	LVEF, mean ± SD (%) I/C
Taghiyev et al. (25)	80	40/40	79.6/78.3	26 (65.0)/32 (80.0)	NA	NA	NA	55.5 ± 9.2/52.4 ± 11.5
Kedhi et al. (26)	172	91/81	76.3/76.6	59 (64.8)/59 (72.8)	69 (75.8)/65 (80.2)	27 (29.6)/27 (33.3)	15 (16.5)/15 (18.5)	54.6 ± 9.4/54.4 ± 8.8
Jagadeesan et al. (27)	37,822	17,413/20,409	77.1/72.7	11,192 (64.3)/15,734 (77.1)	NA	7,574 (43.5)/8,533 (41.8)	5,715 (32.8)/4,839 (23.7)	NA
Amat-Santos et al. (28)	508	254/254	77.9/77.4	88 (34.6)/80 (31.5)	NA	118 (46.5)/114 (44.9)	99 (39.0)/51 (20.1)	55.2 ± 12.7/57.1 ± 11.0
Ullah et al. (29)	11,361	5,358/6,003	79.6/70.2	2,357 (44.0)/394 (6.6)	4,755 (88.7)/5,088 (84.8)	842 (15.7)/1,043 (17.4)	2,176 (40.6)/573 (9.5)	NA
McInerney et al. (30)	1,548	774/774	79.7/80.3	493 (63.7)/439 (56.7)	446 (57.6)/391 (50.5)	318 (41.1)/348 (45)	155 (20.0)/226 (29.2)	NA
Lérault et al. (31)	241	150/91	84.0/73.0	89 (59.3)/67 (73.6)	115 (76.7)/75 (82.4)	61 (40.7)/46 (50.5)	NA	60.0 ± 11.9/60 ± 8.2
Elderia et al. (32)	478	237/241	81.4/71.9	140 (59.1)/185 (76.8)	220 (92.8)/228 (94.6)	76 (32.0)/95 (39.4)	41 (17.2)/48 (19.9)	NA
Alperi et al. (33)	312	156/156	79.5/79.0	90 (66.5)/85 (64)	167 (84.8)/163 (82.7)	72 (46.2)/63 (40.4)	NA	52.1 ± 13.2/52.9 ± 12.9
Søndergaard et al. (34)	332	169/163	79.5	NA	NA	NA	NA	NA
Baumbach et al. (35)	576	112/464	81.3/78.7	NA	73 (65.2)/296 (63.8)	33 (29.5)/180 (38.8)	56 (51.1)/123 (26.5)	51.3 ± 2.6/55.7 ± 13.8
Barbanti et al. (36)	472	236/236	80.7/80.5	128 (54.2)/132 (55.9)	NA	77 (32.6)/80 (33.9)	NA	NA
Wendt et al. (37)	243	59/184	80.0/75.0	26 (44.1)/84(45.7)	NA	19(32.2)/36 (19.6)	NA	49.4 ± 13.0/53.4 ± 12.5

I, intervention group; C, control group; NA, not available; CKD, chronic kidney disease; LVEF, left ventricular ejection fraction; SD, standard deviation.

The two RCTs were assessed with the Cochrane Collaboration's Risk of Bias tool and showed low risk of bias, while the 11 cohort studies scored 7–9 on the NOS, indicating high quality.

### 3.2 Short-term outcomes

#### 3.2.1 30-day all-cause mortality

**Figure 2:**
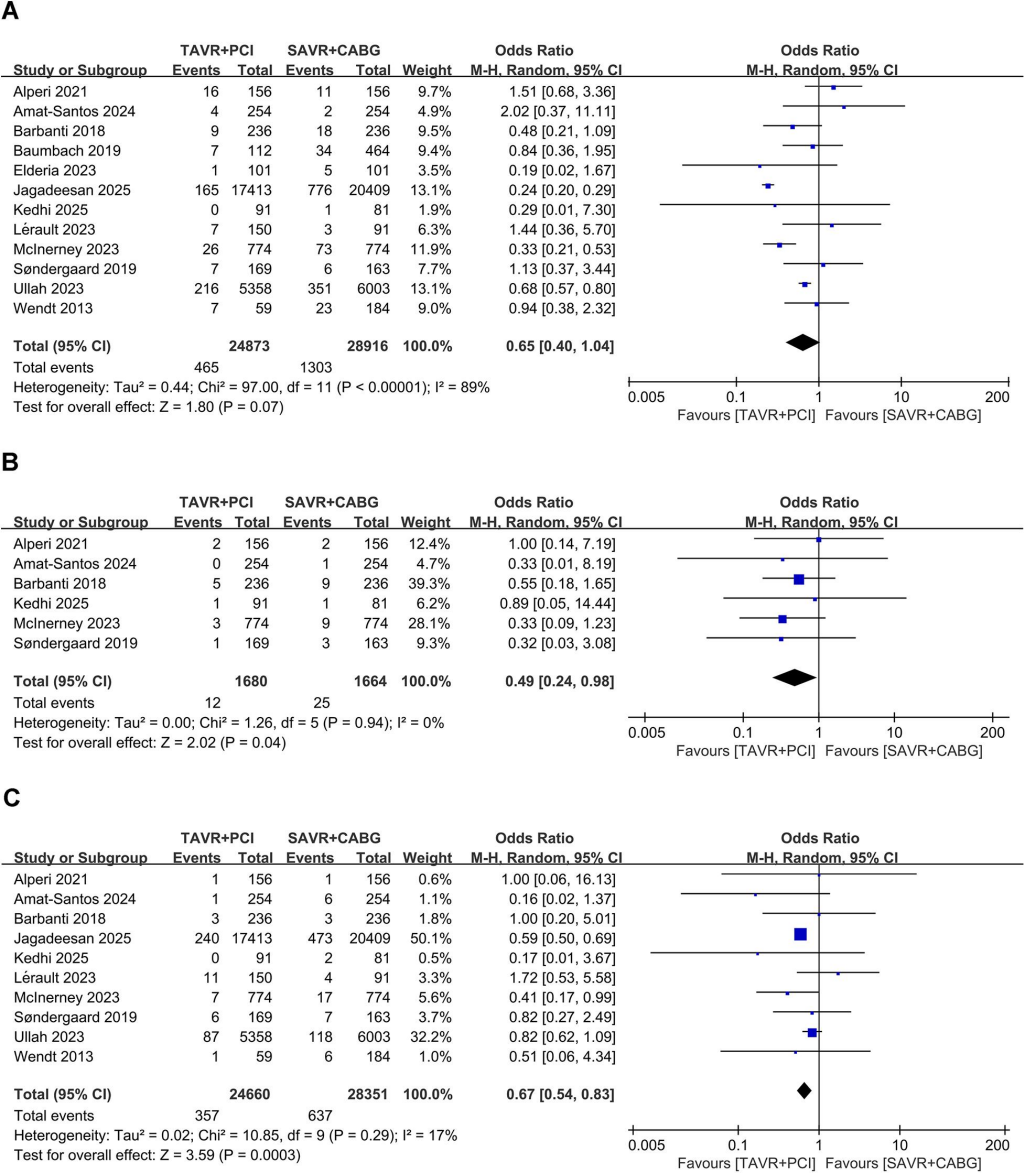
Forest plots for short-term clinical outcomes in intermediate- and high-risk patients undergoing TAVR + PCI vs. SAVR + CABG. **(A)** 30-day all-cause mortality, **(B)** 30-day myocardial infarction, and **(C)** 30-day stroke.

Twelve studies involving 53,789 patients (TAVR + PCI: 24,873; SAVR + CABG: 28,916) were analyzed. Pooled analysis showed no significant difference between groups (OR, 0.65; 95% CI: 0.40–1.04, *P* = 0.07) with substantial heterogeneity (*I*^2^ = 89%, *P* < 0.00001) (Figure 2A). Funnel plot analysis indicated symmetry (Supplementary Figure S2A), and Egger's test (*P* = 0.270) did not suggest significant publication bias.

#### 3.2.2 30-day myocardial infarction

Six studies involving 3,344 patients (TAVR + PCI: 1,680; SAVR + CABG: 1,644) were analyzed. Pooled analysis showed a significantly lower risk of 30-day myocardial infarction in the TAVR + PCI group (OR, 0.49; 95% CI: 0.24–0.98, *P* = 0.04) with no heterogeneity (*I*^2^ = 0%, *P* = 0.94) (Figure 2B). Funnel plot analysis indicated symmetry (Supplementary Figure S2B), and Egger's test (*P* = 0.811) suggested no publication bias.

#### 3.2.3 30-day stroke

Ten studies involving 53,011 patients (TAVR + PCI: 24,660; SAVR + CABG: 28,351) were analyzed. The risk of 30-day stroke was significantly reduced in the TAVR + PCI group compared with SAVR + CABG (OR, 0.67; 95% CI: 0.54–0.83, *P* = 0.0003), with low heterogeneity (*I*^2^ = 17%, *P* = 0.29) (Figure 2C). Funnel plot analysis demonstrated good symmetry (Supplementary Figure S2C), and Egger's test (*P* = 0.890) indicated no significant publication bias.

#### 3.2.4 30-day acute kidney injury

**Figure 3:**
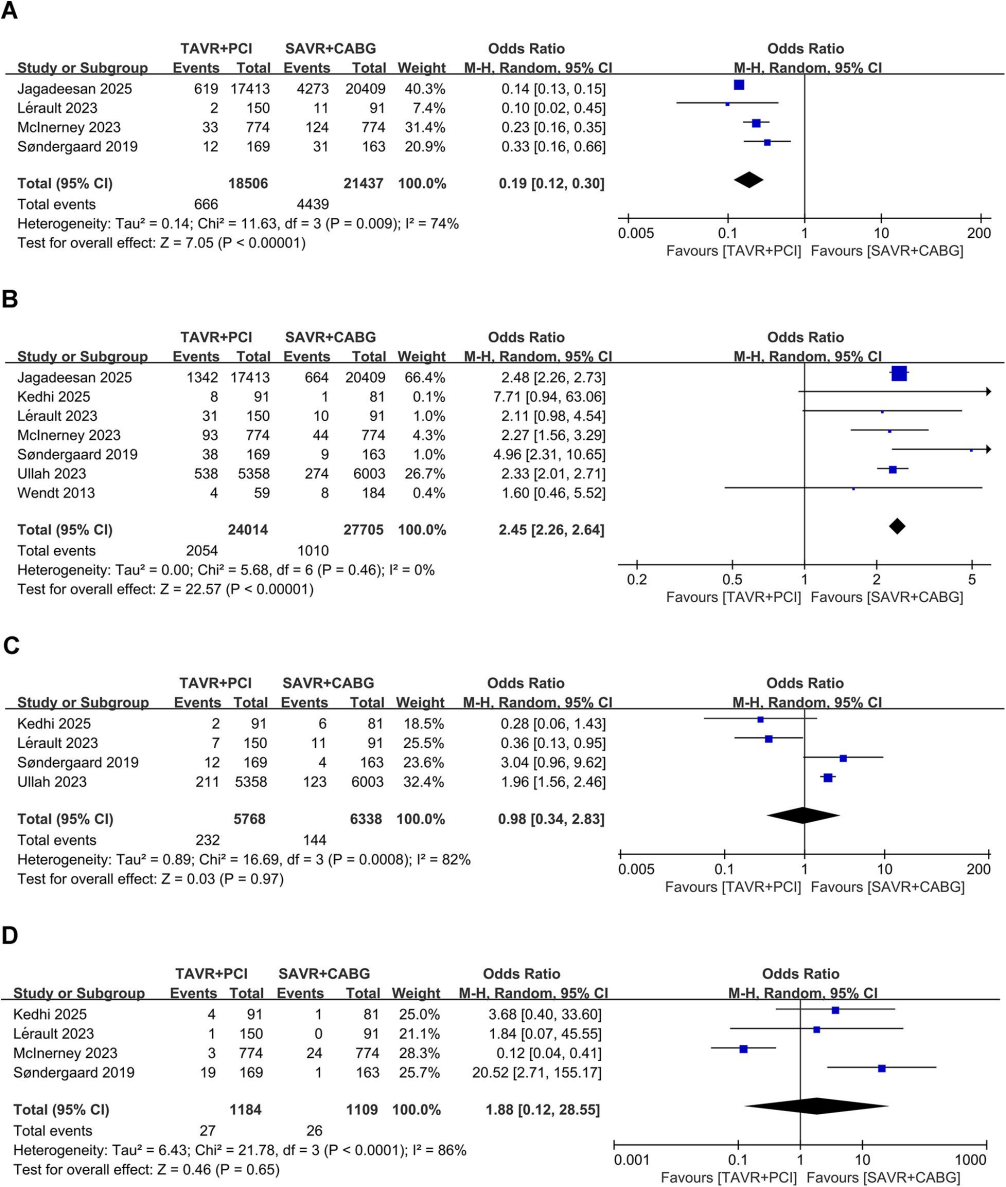
Forest plots for short-term procedural complications in intermediate- and high-risk patients undergoing TAVR + PCI vs. SAVR + CABG. **(A)** 30-day acute kidney injury, **(B)** 30-day permanent pacemaker implantation, **(C)** 30-day major bleeding, and **(D)** 30-day major vascular complications.

Four studies involving 39,943 patients (TAVR + PCI: 18,506; SAVR + CABG: 21,437) were analyzed. TAVR + PCI was associated with a significantly lower incidence of 30-day acute kidney injury compared with SAVR + CABG (OR, 0.19; 95% CI: 0.12–0.30, *P* < 0.00001), although substantial heterogeneity existed (*I*^2^ = 74%, *P* = 0.009) (Figure 3A). Funnel plot analysis suggested symmetry (Supplementary Figure S3A), and Egger's test (*P* = 0.326) did not indicate significant publication bias.

#### 3.2.5 30-day permanent pacemaker implantation

Seven studies involving 51,719 patients (TAVR + PCI: 24,014, SAVR + CABG: 27,705) were analyzed. Pooled analysis showed a significantly higher 30-day permanent pacemaker implantation rate in the TAVR + PCI group (OR, 2.45; 95% CI: 2.26–2.64, *P* < 0.00001), with no heterogeneity (*I*^2^ = 0%, *P* = 0.46) (Figure 3B). Funnel plot showed symmetry (Supplementary Figure S3B), and Egger's test (*P* = 0.586) suggested no significant publication bias.

#### 3.2.6 30-day major bleeding

Four studies involving 12,106 patients (TAVR + PCI: 5,768, SAVR + CABG: 6,338) were analyzed. The pooled analysis showed no significant difference in the risk of 30-day major bleeding between TAVR + PCI and SAVR + CABG (OR, 0.98; 95% CI: 0.34–2.83; *P* = 0.97), although substantial heterogeneity was observed (Figure 3C). Funnel plot showed symmetry (Supplementary Figure S3C), and Egger's test (*P* = 0.336) indicated no significant publication bias.

#### 3.2.7 30-day major vascular complications

Four studies involving 2,293 patients (TAVR + PCI: 1,184, SAVR + CABG: 1,109) were analyzed. Pooled results showed no significant difference between groups (OR, 1.88; 95% CI: 0.12–28.55, *P* = 0.65), with substantial heterogeneity (*I*^2^ = 74%, *P* < 0.001) (Figure 3D). Funnel plot showed symmetry (Supplementary Figure S3D), and Egger's test (*P* = 0.269) suggested no significant publication bias.

### 3.3 Mid- to long-term outcomes

#### 3.3.1 All-cause mortality during follow-up

**Figure 4:**
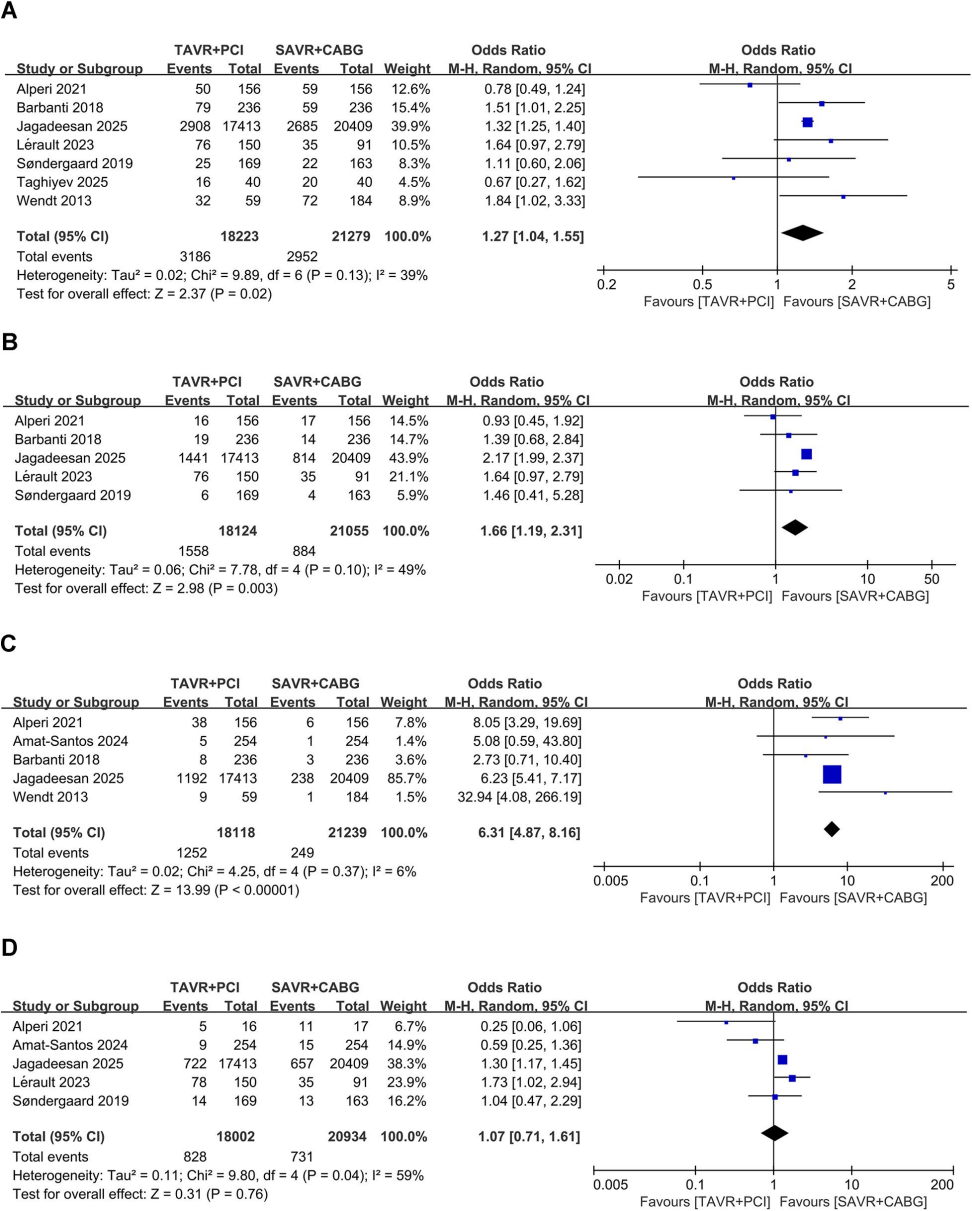
Forest plots for mid- to long-term outcomes in intermediate- and high-risk patients undergoing TAVR + PCI vs. SAVR + CABG. **(A)** All-cause mortality during follow-up, **(B)** myocardial infarction during follow-up, **(C)** revascularization during follow-up, and **(D)** stroke during follow-up.

Seven studies that included 39,502 patients (TAVR + PCI: 18,223, SAVR + CABG: 21,279) were analyzed. Pooled analysis showed higher all-cause mortality during follow-up in the TAVR + PCI group (OR, 1.27; 95% CI: 1.04–1.55, *P* = 0.02), with low heterogeneity (*I*^2^ = 39%, *P* = 0.13) (Figure 4A). Funnel plot appeared symmetrical (Supplementary Figure S4A), and Egger's test revealed no evidence of significant publication bias (*P* = 0.616).

#### 3.3.2 Myocardial infarction during follow-up

Five studies including 39,179 patients (TAVR + PCI: 18,124, SAVR + CABG: 21,055) were analyzed. Pooled analysis showed a significantly higher risk of myocardial infarction during follow-up in the TAVR + PCI group (OR, 1.66; 95% CI: 1.19–2.31, *P* = 0.003), with low heterogeneity (*I*^2^ = 49%, *P* = 0.10) (Figure 4B). However, funnel plot asymmetry was observed (Supplementary Figure S4B), and Egger's test (*P* = 0.013) indicated significant publication bias.

#### 3.3.3 Revascularization during follow-up

Four studies that included 38,849 patients (TAVR + PCI: 17,864, SAVR + CABG: 20,985) were analyzed. Pooled analysis showed a significantly higher revascularization incidence during follow-up in the TAVR + PCI group (OR, 6.49; 95% CI: 4.08–10.33, *P* < 0.00001), with no significant heterogeneity (*I*^2^ = 29%, *P* = 0.24) (Figure 4C). The funnel plot appeared symmetrical (Supplementary Figure S4C), and Egger's test (*P* = 0.772) indicated no substantial publication bias.

#### 3.3.4 Stroke during follow-up

Five studies involving 38,936 patients (TAVR + PCI: 18,002, SAVR + CABG: 20,934) were analyzed. Pooled analysis indicated no significant difference in stroke incidence during follow-up between TAVR + PCI and SAVR + CABG (OR, 1.23; 95% CI 0.88–1.73; *P* = 0.23), with moderate heterogeneity (*I*^2^ = 44%, *P* = 0.15) (Figure 4D). Funnel plot showed no apparent asymmetry (Supplementary Figure S4D), and Egger's test indicated no significant publication bias (*P* = 0.27).

### 3.4 Subgroup analyses

Subgroup analyses were conducted based on prespecified variables, including study design (randomized controlled trials vs. observational studies), sample size (<300 vs. ≥300 participants), and follow-up duration (<5 years vs. ≥5 years). For mid- to long-term all-cause mortality, myocardial infarction, and stroke, a significantly higher risk associated with TAVR + PCI was observed in studies with ≥5 years of follow-up, indicating a potential time-dependent trend. In the analysis of 30-day major bleeding, studies with smaller sample sizes favored TAVR + PCI, whereas larger studies showed the opposite effect, suggesting a possible small-study bias. Detailed results of these subgroup analyses are presented in Table 3 (short-term outcomes) and Table 4 (mid- to long-term outcomes). For other outcomes with high statistical heterogeneity—such as 30-day all-cause mortality, acute kidney injury, and major vascular complications—no consistent subgroup effects were observed across prespecified variables. Although several included studies reported the timing of PCI relative to TAVR, the intervals varied substantially—from a few days to over 12 months—and were inconsistently reported. This procedural heterogeneity likely reflects differences in clinical presentation and institutional protocols, and precluded reliable stratified analysis based on revascularization timing. Similarly, coronary anatomical complexity (e.g., SYNTAX score, left main disease) was not consistently reported across studies, limiting our ability to assess subgroup effects by lesion burden.

**Table 3:** Subgroup analysis of short-term outcomes in intermediate- and high-risk patients undergoing TAVR + PCI vs. SAVR + CABG.

Subgroup	30-day all-cause mortality				30-day myocardial infarction				30-day stroke				30-day major vascular complications			
	Study	OR (95%CI)	*P* value	*I* ^2^	Study	OR (95%CI)	*P* value	*I* ^2^	Study	OR (95%CI)	*P* value	*I* ^2^	Study	OR (95%CI)	*P* value	*I* ^2^
Total	12	0.65 (0.40,1.04)	0.07	89%	6	0.49 (0.24,0.98)	0.04	0%	10	0.67 (0.54,0.83)	0.0003	17%	4	1.88 (0.12,18.18)	0.65	86%
Study design																
RCT	2	0.98 (0.34,2.80)	0.97	0%	2	0.48 (0.08,2.79)	0.41	0%	2	0.68 (0.24,1.94)	0.48	0%	2	9.20 (1.63,52.03)	0.01	26%
Cohort	10	0.63 (0.38,1.04)	0.07	90%	4	0.49 (0.23,1.05)	0.07	0%	8	0.67 (0.52,0.87)	0.003	30%	2	0.31 (0.02,3.88)	0.36	59%
Sample size																
<300 participants	4	0.84 (0.42,1.69)	0.63	0%	1	0.89 (0.05,14.44)	0.93	/	3	0.92 (0.28,3.02)	0.89	19%	2	2.94 (0.48,18.18)	0.25	0%
≥300 participants	8	0.63 (0.37,1.07)	0.09	92%	5	0.47 (0.23,0.96)	0.04	0%	7	0.65 (0.53,0.81)	<0.0001	18%	2	1.48 (0.01,271.89)	0.88	95%
Subgroup	30-day acute kidney injury				30-day major bleeding				30-day permanent pacemaker implantation							
	Study	OR (95% CI)	*P* value	*I* ^2^	Study	OR (95% CI)	*P* value	*I* ^2^	Study	OR (95% CI)	*P* value	*I* ^2^				
Total	4	0.19 (0.12,0.30)	<0.00001	74%	4	0.98 (0.34,2.83)	0.97	82%	7	2.45 (2.26,2.64)	<0.00001	0%				
Study design																
RCT	1	0.33 (0.16,0.66)	0.002	/	2	0.99 (0.10,10.21)	1.00	82%	2	5.22 (2.55,10.70)	<0.00001	0%				
Cohort	3	0.17 (0.11,0.26)	<0.00001	69%	2	0.90 (0.17,4.74)	0.90	91%	5	2.42 (2.24,2.62)	<0.00001	0%				
Sample size																
<300 participants	1	0.10 (0.12,0.30)	<0.00001	74%	2	0.33 (0.14,0.78)	0.01	0%	3	2.20 (1.18,4.11)	0.01	0%				
≥300 participants	3	0.20 (0.12,0.34)	<0.00001	82%	2	1.99 (1.60,2.48)	<0.00001	0%	4	2.45(2.19,2.73)	<0.00001	24%				

OR, odds ratio; CI, confidence interval; RCT, randomized controlled trial; I², heterogeneity index.

**Table 4:** Subgroup analysis of Mid- to long-term outcomes in intermediate- and high-risk patients undergoing TAVR + PCI vs. SAVR + CABG.

Subgroup	All-cause mortality during follow-up				Myocardial infarction during follow-up			
	Study	OR (95% CI)	*P* value	*I* ^2^	Study	OR (95% CI)	*P* value	*I* ^2^
Total	7	1.27 (1.04,1.55)	0.02	39%	5	1.66 (1.19,2.31)	0.003	49%
Follow-up								
<5 years	3	1.24 (0.78,1.96)	0.36	66%	3	1.36 (0.94,1.96)	0.10	0%
≥5 years	4	1.30 (1.05,1.61)	0.02	21%	2	2.17 (1.98,2.37)	<0.00001	0%
Sample size								
<300 participants	3	1.39 (0.83,2.33)	0.21	47%	1	1.64 (0.97,2.79)	0.07	/
≥300 participants	4	1.22 (0.96,1.54)	0.10	47%	4	1.58 (1.00,2.49)	0.05	56%
Subgroup	Stroke during follow-up				Revascularization during follow-up			
	Study	OR (95% CI)	*P* value	*I* ^2^	Study	OR (95% CI)	*P* value	*I* ^2^
Total	4	1.23 (0.88,1.73)	0.23	44%	4	6.49 (4.08,10.33)	<0.00001	29%
Follow-up								
<5 years	2	0.95 (0.25,3.62)	0.94	80%	2	5.28 (1.87,14.90)	0.002	43%
≥5 years	2	1.30 (1.16,1.44)	<0.00001	0%	2	10.20 (2.30,45.27)	0.002	59%
Sample size								
<300 participants	/	/	/	/	1	32.94 (4.08,266.19)	0.001	/
≥300 participants	/	/	/	/	3	6.21 (5.41,7.13)	<0.00001	0%

OR, odds ratio; CI, confidence interval; *I*^2^, heterogeneity index.

### 3.5 Sensitivity analyses

Sensitivity analyses were conducted using leave-one-out procedures to assess the robustness of the pooled estimates. For most outcomes, sequential exclusion of individual studies yielded consistent results, indicating overall stability. An exception was observed in the analysis of 30-day major vascular complications, where omission of the study by McInerney et al. (30) substantially increased the pooled effect size (OR, 6.83; 95% CI: 1.61–28.95), suggesting high sensitivity to this study. Additional sensitivity tests were performed by excluding studies with disproportionately high weights or potential concerns regarding cohort overlap or inconsistent endpoint definitions. These analyses demonstrated that the direction and magnitude of treatment effects remained largely unchanged, supporting the reliability of the overall findings. The full leave-one-out sensitivity results are presented in Supplementary Figures S5–S7.

### 3.6 GRADE evidence quality assessment

This meta-analysis assessed 11 clinical outcomes across short- and mid- to long-term endpoints. Overall GRADE evidence quality ranged from very low to moderate. Evidence certainty for 30-day permanent pacemaker implantation and revascularization during follow-up was rated as moderate, mainly due to a substantial effect size, with no major concerns about bias, inconsistency, or imprecision. For other outcomes, evidence certainty ranged from very low to low. Evidence for 30-day all-cause mortality, 30-day myocardial infarction, 30-day stroke, and 30-day major bleeding was downgraded due to imprecision, while evidence for 30-day acute kidney injury, 30-day major vascular complications, and myocardial infarction during follow-up was downgraded due to serious inconsistency. Additionally, myocardial infarction during follow-up was affected by publication bias (Supplementary Table S6).

## 4 Discussion

This meta-analysis included 13 studies with a total of 53,869 intermediate- and high-risk individuals with severe AS and concomitant CAD. The analysis systematically compared short- and mid- to long-term outcomes between TAVR + PCI and SAVR + CABG. In the short term, TAVR + PCI was associated with lower risks of stroke, myocardial infarction, and acute kidney injury, with no significant difference in all-cause mortality. However, the incidence of permanent pacemaker implantation was notably higher. Over mid- to long-term follow-up (≥2 years), TAVR + PCI was associated with increased risks of all-cause mortality, myocardial infarction, and repeat revascularization, whereas stroke risk remained comparable. Subgroup analyses confirmed the short-term advantages of TAVR + PCI across study designs and sample size stratifications. However, in studies with extended follow-up durations (≥5 years), TAVR + PCI showed a persistently higher risk of all-cause mortality and myocardial infarction during follow-up, raising concerns regarding mid- to long-term durability. The GRADE assessment indicated that evidence quality ranged from very low to moderate.

These short-term findings are consistent with the PARTNER 2 (38) and SURTAVI (2) trials, which demonstrated comparable short-term mortality between TAVR and SAVR, with reduced stroke incidence favoring TAVR. However, these trials did not specifically address concomitant CAD requiring revascularization. By incorporating evidence from RCTs and high-quality cohort studies, the present analysis further supports the short-term safety benefits of TAVR + PCI. Similar results were reported by Sakurai et al. (14), who identified no significant difference in 30-day mortality but lower acute kidney injury and higher pacemaker implantation rates with TAVR + PCI. Notably, that analysis lacked stratification by surgical risk, potentially contributing to heterogeneity. By focusing specifically on intermediate- and high-risk individuals, this meta-analysis additionally identified reduced risks of stroke and myocardial infarction with TAVR + PCI, findings not clearly emphasized in previous meta-analyses. Among the included studies, the TCW trial by Kedhi et al. (26) provided RCT-level evidence supporting favorable short-term outcomes with TAVR + PCI in elderly patients with anatomically suitable CAD, though limited by short follow-up and exclusion of patients with complex coronary anatomy. The short-term benefits of TAVR + PCI may be attributed to its minimally invasive nature, avoiding cardiopulmonary bypass and reducing procedural trauma, thereby lowering the risks of stroke, hypoperfusion-related injury, and acute kidney injury. PCI also facilitates timely revascularization, potentially reducing perioperative myocardial infarction. The increased rate of permanent pacemaker implantation following TAVR is likely related to atrioventricular node injury, particularly with self-expanding valves, as documented in prior studies.

Regarding mid- to long-term outcomes, the present findings align with the EXCEL (39) and SYNTAX (40) trials, which demonstrated superior mid- to long-term outcomes with CABG compared to PCI in patients with complex CAD. Although these trials did not include patients undergoing aortic valve intervention, the current analysis extends their conclusions to a high-risk AS population. The increased rates of myocardial infarction and repeat revascularization observed with TAVR + PCI may reflect incomplete revascularization or disease progression in untreated segments. In contrast, CABG provides durable distal perfusion via bypass grafts, which may contribute to mid- to long-term myocardial protection. Valve durability may also influence mid- to long-term outcomes. While early-generation TAVR valves showed structural deterioration within 5–8 years, surgical bioprosthetic valves typically last 10–15 years (41). However, more recent studies (e.g., PARTNER 2/3 and FRANCE-TAVI studies) (38, 42, 43) suggest that newer transcatheter valves exhibit improved durability, though further validation through mid- to long-term follow-up is necessary. Paravalvular leak following TAVR may lead to chronic volume overload, potentially affecting ventricular remodeling and mid- to long-term survival. Despite the worse mid- to long-term outcomes, stroke incidence during follow-up did not differ significantly between groups, possibly reflecting differences in antithrombotic strategies, baseline atrial fibrillation burden, and sample size.

Importantly, interpretation of these findings should be contextualized within the framework of the 2025 ESC/EACTS Guidelines (6) for the management of valvular heart disease, which emphasize individualized, Heart Team–based decision-making that integrates clinical risk, anatomical complexity, imaging findings, and patient preference, rather than relying solely on chronological age or surgical risk scores. In this context, the present results underscore that while TAVR + PCI may offer short-term procedural advantages, particularly in frail or high-risk patients, it may be associated with less favorable mid- to long-term outcomes in individuals with complex coronary anatomy or a longer life expectancy. Accordingly, patients with multivessel or anatomically complex CAD, in whom complete revascularization is more reliably achieved with CABG, may derive greater mid- to long-term benefit from SAVR + CABG, provided surgical risk is acceptable. Conversely, it is equally important to recognize clinical scenarios in which TAVR + PCI may not be recommended or technically feasible, including unfavorable annular or aortic root anatomy, inadequate vascular access, the need for concomitant surgical procedures (e.g., ascending aortic repair or other valve interventions), or specific valve morphologies such as certain bicuspid aortic valve phenotypes. These considerations reinforce that TAVR + PCI should not be viewed as a universal substitute for surgery but rather as one component of a tailored treatment strategy. At the same time, surgical treatment may be infeasible in selected patient subsets—such as those with porcelain aorta, prohibitive redo sternotomy risk, or advanced frailty—in whom TAVR + PCI may represent a feasible, and in some cases the only practical, option for combined valve intervention and coronary revascularization. The present findings therefore highlight the necessity of balancing early procedural safety against longer-term risks when selecting revascularization and valve strategies in this complex population.

This meta-analysis provides an updated and comprehensive synthesis comparing TAVR + PCI and SAVR + CABG in this intermediate- and high-risk patients with severe AS and concomitant CAD. Methodological rigor—including adherence to PRISMA guidelines, GRADE assessment, and pre-specified subgroup and sensitivity analyses—strengthens the robustness of the findings. Nonetheless, several limitations warrant discussion. First, substantial heterogeneity was observed across included studies in terms of patient characteristics, procedural details (e.g., valve type, access route), and operator experience, which may have influenced effect estimates. Second, PCI timing relative to TAVR varied widely, ranging from staged to simultaneous procedures, and was inconsistently reported, limiting the feasibility of stratified analyses by timing. Third, detailed coronary anatomical data (e.g., SYNTAX score, left main disease) were inconsistently reported, constraining lesion-complexity–based comparisons. Fourth, although HRs were prespecified as the preferred effect measure for mid- to long-term outcomes, most included studies only reported crude event counts and did not provide HRs or extractable Kaplan–Meier curves. Consequently, ORs derived from event numbers were used as an alternative, which may not fully capture the temporal dimension of outcomes and could have influenced the precision of mid- to long-term risk estimates. Fifth, outcome definitions were not fully standardized across studies (e.g., some applied VARC-2 or VARC-3 criteria, while others did not specify criteria). Although we systematically summarized all reported definitions in Supplementary Tables S3 and S4, such inconsistency may have contributed to residual heterogeneity, particularly for revascularization, where most studies did not distinguish target-lesion vs. any-vessel events, planned vs. unplanned staged procedures, or graft-failure–related reinterventions. Sixth, while procedural and anatomical effect modifiers (e.g., access route, valve type, PCI timing, completeness of revascularization, and SYNTAX strata) were considered *a priori*, their implementation was limited by non-uniform reporting and lack of harmonized definitions across studies; as a result, only consistently available subgroup variables—follow-up duration (<5 vs. ≥5 years) and study size (<300 vs. ≥300)—could be analyzed uniformly, with study design explored where feasible. Seventh, although two RCTs were included, the evidence base was predominantly derived from observational studies and therefore remains vulnerable to selection bias and residual confounding, even when adjustment techniques—such as propensity score matching or multivariable regression—were applied at the individual study level in some cohorts. Lastly, the overall certainty of evidence ranged from low to very low, primarily due to inconsistency and imprecision, underscoring the need for additional high-quality data. In the context of the 2025 ESC/EACTS Guidelines, these limitations highlight the need for cautious interpretation when extrapolating comparative effectiveness to individual clinical decision-making. Future research should therefore prioritize well-powered randomized trials with extended follow-up, standardized time-to-event outcome reporting, and stratification by coronary anatomy and clinical risk. Equally important, harmonized reporting of procedural and anatomical variables—including access route, valve type, PCI timing with explicit staged vs. concomitant definitions, completeness of revascularization, and quantitative SYNTAX categories—as well as consistent adoption of standardized outcome definitions (e.g., VARC-3), will be essential to enhance comparability across studies. Meta-analyses incorporating patient-level data and guideline-aligned outcome frameworks may further improve evidence quality and clinical applicability.

## 5 Conclusion

TAVR + PCI is associated with lower early periprocedural complications but may carry higher mid- to long-term risks compared with SAVR + CABG, although the certainty of evidence is limited by observational data and heterogeneity. Treatment decisions should be individualized, considering CAD complexity, revascularization completeness, valve type, and procedural timing. These findings underscore the importance of balancing early procedural safety against potential mid- to long-term risks when selecting treatment strategies in patients with severe AS and concomitant CAD. Further randomized trials with standardized outcome definitions and extended follow-up are warranted.

## Data Availability

The original contributions presented in the study are included in the article/supplementary material, further inquiries can be directed to the corresponding author/s.
