# Supplementary material for: Comparing TAVR + PCI vs. SAVR + CABG across short- and mid- to long-term horizons in patients with severe aortic stenosis and concomitant CAD: a systematic review and meta-analysis
Source: Front Cardiovasc Med. 2026 Jan 30;13:1640906. doi: 10.3389/fcvm.2026.1640906 (PMC12901452; doi:10.3389/fcvm.2026.1640906)
Supplement: Supplementary file 1 [file Datasheet1.docx]

**Supplementary Materials**

**Supplementary Tables**

**Supplementary Table S1.** PRISMA 2020 Checklist for Reporting Systematic Reviews and Meta-Analyses.

**Supplementary Table S2.** Literature search strategy.

**Supplementary Table S3.** Definitions of Short-Term Outcomes in Included Studies.

**Supplementary Table S4.** Definitions of Mid- to Long-Term Outcomes in Included Studies.

**Supplementary Table S5.** Newcastle-Ottawa Scale Quality Assessment for Included Cohort Studies.

**Supplementary Table S6.** GRADE Assessment of Evidence Quality for Outcomes.

**Supplementary Figures**

**Supplementary Figure S1.** Risk of Bias Assessment for RCTs.

**Supplementary Figure S2.** Funnel Plots for Publication Bias in Short-Term Outcomes in Intermediate- and High-Risk Patients Undergoing TAVR+PCI vs. SAVR+CABG.

**Supplementary Figure S3.** Funnel Plots for Publication Bias in Short-Term Procedural Complications in Intermediate- and High-Risk Patients Undergoing TAVR+PCI vs. SAVR+CABG.

**Supplementary Figure S4.** Funnel Plots for Publication Bias in Mid- to Long-Term Outcomes in Intermediate- and High-Risk Patients Undergoing TAVR+PCI vs. SAVR+CABG.

**Supplementary Figure S5.** Sensitivity Analysis for Short-Term Outcomes in Intermediate- and High-Risk Patients Undergoing TAVR+PCI vs. SAVR+CABG.

**Supplementary Figure S6.** Sensitivity Analysis for Short-Term Procedural Complications in Intermediate- and High-Risk Patients Undergoing TAVR+PCI vs. SAVR+CABG.

**Supplementary Figure S7.** Sensitivity Analysis for Mid- to Long-Term Outcomes in Intermediate- and High-Risk Patients Undergoing TAVR+PCI vs. SAVR+CABG.

**Supplementary Table S1.** PRISMA 2020 Checklist for Reporting Systematic Reviews and Meta-Analyses.

| **Section and Topic** | **Item #** | **Checklist item** | **Location where item is reported** |
| --- | --- | --- | --- |
| **TITLE** | | |  |
| Title | 1 | Identify the report as a systematic review. | Title |
| **ABSTRACT** | | |  |
| Abstract | 2 | See the PRISMA 2020 for Abstracts checklist. | Abstract |
| **INTRODUCTION** | | |  |
| Rationale | 3 | Describe the rationale for the review in the context of existing knowledge. | Introduction |
| Objectives | 4 | Provide an explicit statement of the objective(s) or question(s) the review addresses. | Introduction |
| **METHODS** | | |  |
| Eligibility criteria | 5 | Specify the inclusion and exclusion criteria for the review and how studies were grouped for the syntheses. | Methods |
| Information sources | 6 | Specify all databases, registers, websites, organisations, reference lists and other sources searched or consulted to identify studies. Specify the date when each source was last searched or consulted. | Methods |
| Search strategy | 7 | Present the full search strategies for all databases, registers and websites, including any filters and limits used. | Methods |
| Selection process | 8 | Specify the methods used to decide whether a study met the inclusion criteria of the review, including how many reviewers screened each record and each report retrieved, whether they worked independently, and if applicable, details of automation tools used in the process. | Methods |
| Data collection process | 9 | Specify the methods used to collect data from reports, including how many reviewers collected data from each report, whether they worked independently, any processes for obtaining or confirming data from study investigators, and if applicable, details of automation tools used in the process. | Methods |
| Data items | 10a | List and define all outcomes for which data were sought. Specify whether all results that were compatible with each outcome domain in each study were sought (e.g. for all measures, time points, analyses), and if not, the methods used to decide which results to collect. | Methods |
|  | 10b | List and define all other variables for which data were sought (e.g. participant and intervention characteristics, funding sources). Describe any assumptions made about any missing or unclear information. | Methods |
| Study risk of bias assessment | 11 | Specify the methods used to assess risk of bias in the included studies, including details of the tool(s) used, how many reviewers assessed each study and whether they worked independently, and if applicable, details of automation tools used in the process. | Methods |
| Effect measures | 12 | Specify for each outcome the effect measure(s) (e.g. risk ratio, mean difference) used in the synthesis or presentation of results. | Methods |
| Synthesis methods | 13a | Describe the processes used to decide which studies were eligible for each synthesis (e.g. tabulating the study intervention characteristics and comparing against the planned groups for each synthesis (item #5)). | Methods |
|  | 13b | Describe any methods required to prepare the data for presentation or synthesis, such as handling of missing summary statistics, or data conversions. | Methods |
|  | 13c | Describe any methods used to tabulate or visually display results of individual studies and syntheses. | Methods |
|  | 13d | Describe any methods used to synthesize results and provide a rationale for the choice(s). If meta-analysis was performed, describe the model(s), method(s) to identify the presence and extent of statistical heterogeneity, and software package(s) used. | Methods |
|  | 13e | Describe any methods used to explore possible causes of heterogeneity among study results (e.g. subgroup analysis, meta-regression). | Methods |
|  | 13f | Describe any sensitivity analyses conducted to assess robustness of the synthesized results. | Methods |
| Reporting bias assessment | 14 | Describe any methods used to assess risk of bias due to missing results in a synthesis (arising from reporting biases). | Methods |
| Certainty assessment | 15 | Describe any methods used to assess certainty (or confidence) in the body of evidence for an outcome. | Methods |
| **RESULTS** | | |  |
| Study selection | 16a | Describe the results of the search and selection process, from the number of records identified in the search to the number of studies included in the review, ideally using a flow diagram. | Results |
|  | 16b | Cite studies that might appear to meet the inclusion criteria, but which were excluded, and explain why they were excluded. | Results |
| Study characteristics | 17 | Cite each included study and present its characteristics. | Results |
| Risk of bias in studies | 18 | Present assessments of risk of bias for each included study. | Results |
| Results of individual studies | 19 | For all outcomes, present, for each study: (a) summary statistics for each group (where appropriate) and (b) an effect estimate and its precision (e.g. confidence/credible interval), ideally using structured tables or plots. | Results |
| Results of syntheses | 20a | For each synthesis, briefly summarise the characteristics and risk of bias among contributing studies. | Results |
|  | 20b | Present results of all statistical syntheses conducted. If meta-analysis was done, present for each the summary estimate and its precision (e.g. confidence/credible interval) and measures of statistical heterogeneity. If comparing groups, describe the direction of the effect. | Results |
|  | 20c | Present results of all investigations of possible causes of heterogeneity among study results. | Results |
|  | 20d | Present results of all sensitivity analyses conducted to assess the robustness of the synthesized results. | Results |
| Reporting biases | 21 | Present assessments of risk of bias due to missing results (arising from reporting biases) for each synthesis assessed. | Results |
| Certainty of evidence | 22 | Present assessments of certainty (or confidence) in the body of evidence for each outcome assessed. | Results |
| **DISCUSSION** | | |  |
| Discussion | 23a | Provide a general interpretation of the results in the context of other evidence. | Discussion |
|  | 23b | Discuss any limitations of the evidence included in the review. | Discussion |
|  | 23c | Discuss any limitations of the review processes used. | Discussion |
|  | 23d | Discuss implications of the results for practice, policy, and future research. | Discussion |
| **OTHER INFORMATION** | | |  |
| Registration and protocol | 24a | Provide registration information for the review, including register name and registration number, or state that the review was not registered. | Methods |
|  | 24b | Indicate where the review protocol can be accessed, or state that a protocol was not prepared. | Methods |
|  | 24c | Describe and explain any amendments to information provided at registration or in the protocol. | Methods |
| Support | 25 | Describe sources of financial or non-financial support for the review, and the role of the funders or sponsors in the review. | Declarations |
| Competing interests | 26 | Declare any competing interests of review authors. | Declarations |
| Availability of data, code and other materials | 27 | Report which of the following are publicly available and where they can be found: template data collection forms; data extracted from included studies; data used for all analyses; analytic code; any other materials used in the review. | Declarations |

*From:*  Page MJ, McKenzie JE, Bossuyt PM, Boutron I, Hoffmann TC, Mulrow CD, et al. The PRISMA 2020 statement: an updated guideline for reporting systematic reviews. BMJ 2021;372:n71. doi: 10.1136/bmj.n71

For more information, visit: <http://www.prisma-statement.org/>

# Table S2. Literature search strategy.

# 1.Pubmed

| Search number | Query |
| --- | --- |
| #1 | ((((Transcatheter Aortic Valve Replacement[MeSH Terms]) OR (Transcatheter Aortic Valve Replacement[Title/Abstract])) OR (Transcatheter Aortic Valve Implantation[Title/Abstract])) OR (TAVR[Title/Abstract])) OR (TAVI[Title/Abstract]) |
| #2 | (((((Percutaneous Coronary Intervention[MeSH Terms]) OR (Percutaneous Coronary Intervention[Title/Abstract])) OR (Percutaneous Coronary Interventions[Title/Abstract])) OR (Percutaneous Coronary Revascularization[Title/Abstract])) OR (Coronary Stent[Title/Abstract])) OR (PCI[Title/Abstract]) |
| #3 | (surgical aortic valve replacement[Title/Abstract]) OR (SAVR[Title/Abstract]) |
| #4 | (((((((Coronary Artery Bypass[MeSH Terms]) OR (Coronary Artery Bypass[Title/Abstract])) OR (Coronary Artery Bypasses[Title/Abstract])) OR (Coronary Artery Bypass Grafting[Title/Abstract])) OR (Coronary Artery Bypass Surgery[Title/Abstract])) OR (Aortocoronary Bypass[Title/Abstract])) OR (Aortocoronary Bypasses[Title/Abstract])) OR (CABG[Title/Abstract]) |
| #5 | (((((((Transcatheter Aortic Valve Replacement[MeSH Terms]) OR (Transcatheter Aortic Valve Replacement[Title/Abstract])) OR (Transcatheter Aortic Valve Implantation[Title/Abstract])) OR (TAVR[Title/Abstract])) OR (TAVI[Title/Abstract])) AND ((((((Percutaneous Coronary Intervention[MeSH Terms]) OR (Percutaneous Coronary Intervention[Title/Abstract])) OR (Percutaneous Coronary Interventions[Title/Abstract])) OR (Percutaneous Coronary Revascularization[Title/Abstract])) OR (Coronary Stent[Title/Abstract])) OR (PCI[Title/Abstract]))) AND ((surgical aortic valve replacement[Title/Abstract]) OR (SAVR[Title/Abstract]))) AND ((((((((Coronary Artery Bypass[MeSH Terms]) OR (Coronary Artery Bypass[Title/Abstract])) OR (Coronary Artery Bypasses[Title/Abstract])) OR (Coronary Artery Bypass Grafting[Title/Abstract])) OR (Coronary Artery Bypass Surgery[Title/Abstract])) OR (Aortocoronary Bypass[Title/Abstract])) OR (Aortocoronary Bypasses[Title/Abstract])) OR (CABG[Title/Abstract])) |

**2.the Cochrane library**

| Search number | Query |
| --- | --- |
| #1 | MeSH descriptor: [Transcatheter Aortic Valve Replacement] explode all trees |
| #2 | (Transcatheter Aortic Valve Replacement):ti,ab,kw OR (Transcatheter Aortic Valve Implantation):ti,ab,kw OR (TAVR):ti,ab,kw OR (TAVI):ti,ab,kw (Word variations have been searched) |
| #3 | MeSH descriptor: [Percutaneous Coronary Intervention] explode all trees |
| #4 | (Percutaneous Coronary Intervention):ti,ab,kw OR (Percutaneous Coronary Interventions):ti,ab,kw OR (Percutaneous Coronary Revascularization):ti,ab,kw OR (Coronary Stent):ti,ab,kw OR (PCI):ti,ab,kw (Word variations have been searched) |
| #5 | (surgical aortic valve replacement):ti,ab,kw OR (SAVR):ti,ab,kw (Word variations have been searched) |
| #6 | MeSH descriptor: [Coronary Artery Bypass] explode all trees |
| #7 | (coronary artery bypass):ti,ab,kw OR (Coronary Artery Bypasses):ti,ab,kw OR (Coronary Artery Bypass Grafting):ti,ab,kw OR (Coronary Artery Bypass Surgery):ti,ab,kw OR (Aortocoronary Bypass):ti,ab,kw (Word variations have been searched) |
| #8 | (Aortocoronary Bypasses):ti,ab,kw OR (CABG):ti,ab,kw (Word variations have been searched) |
| #9 | #1 OR #2 |
| #10 | #3 OR #4 |
| #11 | #6 OR #7 OR #8 |
| #12 | #9 AND #10 AND #5 AND #11 |

**3.Embase**

| Search number | Query |
| --- | --- |
| #1 | 'transcatheter aortic valve implantation'/exp |
| #2 | 'transcatheter aortic valve replacement':ab,ti |
| #3 | 'transcatheter aortic valve implantation':ab,ti |
| #4 | 'tavr':ab,ti |
| #6 | #1 OR #2 OR #3 OR #4 OR #5 |
| #7 | 'percutaneous coronary intervention'/exp |
| #8 | 'percutaneous coronary intervention':ab,ti |
| #9 | 'percutaneous coronary interventions':ab,ti |
| #10 | 'percutaneous coronary revascularization':ab,ti |
| #11 | 'coronary stent':ab,ti |
| #12 | 'pci':ab,ti |
| #13 | #7 OR #8 OR #9 OR #10 OR #11 OR #12 |
| #14 | 'surgical aortic valve replacement':ab,ti |
| #15 | 'savr':ab,ti |
| #16 | #14 OR #15 |
| #17 | 'coronary artery bypass graft'/exp |
| #18 | 'coronary artery bypass graft':ab,ti |
| #19 | 'coronary artery bypasses':ab,ti |
| #20 | 'coronary artery bypass grafting':ab,ti |
| #21 | 'coronary artery bypass surgery':ab,ti |
| #22 | 'aortocoronary bypass':ab,ti |
| #23 | 'aortocoronary bypasses':ab,ti |
| #24 | 'cabg':ab,ti |
| #25 | #17 OR #18 OR #19 OR #20 OR #21 OR #22 OR #23 OR #24 |
| #26 | #6 AND #13 AND #16 AND #25 |

**4.Web of science**

| Search number | Query |
| --- | --- |
| #1 | (((TS=(Transcatheter Aortic Valve Replacement )) OR TS=(Transcatheter Aortic Valve Implantation)) OR TS=(TAVR)) OR TS=(TAVI) and Preprint Citation Index (Exclude – Database) |
| #2 | ((((TS=(Percutaneous Coronary Intervention)) OR TS=(Percutaneous Coronary Interventions)) OR TS=(Percutaneous Coronary Revascularization)) OR TS=(Coronary Stent)) OR TS=(PCI) and Preprint Citation Index (Exclude – Database) |
| #3 | (TS=(surgical aortic valve replacement )) OR TS=(SAVR) and Preprint Citation Index (Exclude – Database) |
| #4 | ((((((((TS=(surgical aortic valve replacement )) OR TS=(SAVR)) AND TS=(Coronary Artery Bypass )) OR TS=(Coronary Artery Bypasses)) OR TS=(Coronary Artery Bypass Grafting)) OR TS=(Coronary Artery Bypass Surgery)) OR TS=(Aortocoronary Bypass)) OR TS=(Aortocoronary Bypasses)) OR TS=(CABG) and Preprint Citation Index (Exclude – Database) |
| #5 | #1 AND #2 AND #3 AND #4 and Preprint Citation Index (Exclude – Database) |

**Supplementary Table S3.** Definitions of Short-Term Outcomes in Included Studies.

| **Study** | **30-day all-cause mortality** | **30-day MI** | **30-day stroke** | **30-day major vascular complications** | **30-day AKI** | **30-day major bleeding** | **30-day permanent pacemaker implantation** |
| --- | --- | --- | --- | --- | --- | --- | --- |
| Taghiyev 2025 | NA | NA | NA | NA | NA | NA | NA |
| Kedhi 2025 | Defined as death from any cause occurring within 30 days following the index procedure (TAVR or SAVR), as recorded in hospital records or early follow-up documentation. | Defined according to study protocol. MI was classified as periprocedural or spontaneous. Detailed diagnostic criteria and fatality status were not specified in the published article. | Defined as an acute neurological deficit lasting ≥24 hours, adjudicated by a clinical events committee. Strokes were classified as disabling or non-disabling. Stroke subtype (ischemic vs. hemorrhagic) was not consistently reported across studies. | Defined according to the Valve Academic Research Consortium-2 (VARC-2) criteria, including but not limited to access site bleeding, pseudoaneurysm, dissection, or other vascular injury requiring intervention. | NA | Defined according to the VARC-2 bleeding classification. Major bleeding was defined as overt bleeding with a hemoglobin drop ≥3 g/dL or requiring transfusion or surgical intervention. TCW trial also reported life-threatening and minor events. | Defined as new permanent pacemaker implantation within 30 days of the index procedure, indicated by new-onset conduction disturbances (e.g., atrioventricular block or bundle branch block), as a prespecified safety endpoint in the TCW trial. |
| Jagadeesan 2025 | Defined as death from any cause occurring within 30 days following the index procedure (TAVR or SAVR), based on hospitalization records or early follow-up documentation. | NA | Defined as an acute neurological deficit occurring within 30 days post-procedure, requiring hospitalization and identified via ICD-10 diagnostic codes for ischemic or hemorrhagic stroke (e.g., I63.x, I61.x). | NA | Defined as acute impairment of renal function during the index hospitalization, identified using ICD-10 diagnostic codes (e.g., N17.x). Some studies also incorporated KDIGO staging criteria. | NA | Defined as new permanent pacemaker implantation within 30 days of the index procedure due to new-onset atrioventricular or bundle branch block, verified via procedural coding. |
| Amat-Santos 2024 | Defined as death from any cause occurring within 30 days post-procedure, as reported as “30-day mortality” in both unmatched and matched cohorts. | Defined as myocardial infarction occurring within 30 days post-procedure, identified via hospitalization records. Diagnostic criteria (e.g., ECG, biomarkers) not specified. | Defined as new-onset stroke occurring during index hospitalization or within 30 days post-procedure. Stroke type, duration, and diagnostic confirmation were not reported. | NA | NA | NA | NA |
| Ullah 2023 | Defined as death from any cause during the index hospitalization, including cardiovascular and non-cardiovascular causes. Events occurring within 30 days were identified using admission records in the NIS dataset. | NA | Defined as ischemic or embolic cerebrovascular events confirmed by brain imaging and recorded using diagnostic codes during hospitalization. Reported as part of 30-day outcomes. | NA | NA | Defined as intracavitary or intracranial bleeding, or bleeding requiring blood transfusion, recorded during index hospitalization. | Defined as new permanent pacemaker implantation within 30 days to manage atrioventricular conduction disturbances. Reported overall and stratified by procedural timing (e.g., same-day vs delayed). |
| McInerney 2023 | Defined as death from any cause occurring within 30 days following the index procedure. | Not specifically defined. MI reported in the 30-day outcomes table without diagnostic criteria. | Not specifically defined. Stroke was reported in the 30-day outcome table without further detail. | Not defined. | Not defined. | NA | Defined as new requirement for permanent pacemaker implantation within 30 days post-procedure. No electrophysiological or ECG criteria were specified. |
| Lérault 2023 | Defined as death from any cause occurring within 30 days post-procedure. | NA | Not defined. | Not defined. | Defined according to VARC-3 criteria as stage 2 or 3 AKI, based on a ≥200% increase in serum creatinine. Methods referenced in Section 2.4. | Reported as “life-threatening bleeding” per VARC-3 criteria. No further specification regarding thresholds or classification provided. | Reported as new permanent pacemaker requirement within 30 days. No ECG-based thresholds or conduction block grading was described. |
| Elderia 2023 | Defined as death from any cause during the hospital stay or within 30 days post-procedure, according to VARC-3 recommendations. | NA | NA | NA | NA | NA | NA |
| Alperi 2021 | Defined as death from any cause occurring within 30 days following the index procedure, assessed using hospital records and vital status documentation. | Defined as myocardial infarction classified as STEMI or type 1 NSTEMI based on case report forms. Universal diagnostic criteria (e.g., ECG, biomarker thresholds) were not provided. | Defined as stroke reported with date in the case report form. No information on duration, subtype, or adjudication was provided. | NA | NA | NA | NA |
| Søndergaard 2019 | Defined as death from any cause occurring within 30 days following the index procedure. | Defined and adjudicated according to Valve Academic Research Consortium-2 (VARC-2) criteria. Specific thresholds for ECG or troponin were not reported. | Defined as neurological deficit adjudicated by an independent neurologist, using VARC-2 definitions. Both disabling and non-disabling strokes were included. Stroke subtype was not specified. | Defined according to VARC-2 criteria, including access-site complications requiring medical or surgical intervention. | Defined according to acute kidney injury stages (Stage 1–3). Although KDIGO or AKIN criteria were not explicitly stated, staging implies use of standard definitions. | Defined as “major bleeding” or “life-threatening/disabling bleeding” adjudicated per VARC-2 criteria. | Defined as requirement for new permanent pacemaker implantation during index hospitalization or within 30 days. Clearly documented and compared. |
| Baumbach 2019 | Not defined. | NA | NA | NA | NA | NA | NA |
| Barbanti 2018 | Defined as death from any cause occurring within 30 days following the procedure. | Not specifically defined. | Not defined. | NA | NA | NA | NA |
| Wendt 2013 | Defined as death from any cause occurring within 30 days post-intervention or during the index hospitalization. | NA | Not defined. | NA | NA | NA | Not defined. |

TAVR, transcatheter aortic valve replacement; SAVR, surgical aortic valve replacement; MI, myocardial infarction; NSTEMI, non–ST-elevation myocardial infarction; STEMI, ST-elevation myocardial infarction; ECG, electrocardiogram; ICD-10, International Classification of Diseases, Tenth Revision; VARC-2, Valve Academic Research Consortium-2; VARC-3, Valve Academic Research Consortium-3; KDIGO, Kidney Disease: Improving Global Outcomes; AKI, acute kidney injury; PCI, percutaneous coronary intervention; NIS, National Inpatient Sample; NA, not available.

Short-term outcomes were defined as adverse events occurring within 30 days of the index procedure. Definitions were extracted directly or paraphrased from each original study, retaining alignment with published protocols. Where available, standard criteria (e.g., VARC-2, VARC-3, KDIGO) were cited.

**Supplementary Table S4.** Definitions of Mid- to Long-Term Outcomes in Included Studies.

| **Study** | **All-cause mortality during follow-up (≥ 2 years)** | **Myocardial infarction during follow-up (≥ 2 years)** | **Revascularization during follow-up (≥ 2 years)** | **Stroke during follow-up (≥ 2 years)** |
| --- | --- | --- | --- | --- |
| Taghiyev 2025 | Defined as death from any cause during the post-procedural follow-up period. Mortality was assessed using Kaplan–Meier estimates over a median follow-up of 6.4 years (interquartile range: 3.5–9.2). | NA | NA | NA |
| Kedhi 2025 | NA | NA | NA | NA |
| Jagadeesan 2025 | Defined as death from any cause during the entire post-procedural follow-up. Mortality status was identified using national claims databases (e.g., Medicare), with longitudinal tracking up to 5 years. | Defined as any hospital admission with a primary discharge diagnosis of myocardial infarction during follow-up, identified using ICD-10-CM codes. Subclassification into spontaneous or procedural MI varied across studies. | Defined as any repeat coronary revascularization procedure (PCI or CABG) performed during the follow-up period, identified using procedure or claims codes. Clinically driven revascularizations were included. | Defined as any new ischemic or hemorrhagic stroke resulting in hospitalization during follow-up, identified using diagnostic claims codes. Duration and confirmation of neurological deficit varied among included studies. |
| Amat-Santos 2024 | NA | NA | NA | NA |
| Ullah 2023 | NA | NA | NA | NA |
| McInerney 2023 | NA | NA | NA | NA |
| Lérault 2023 | Defined as death from any cause during follow-up (median 27 months). Events were ascertained from hospital records and physician reports. No adjudication committee was involved. | Not defined. | NA | Reported as stroke events during follow-up, without specification of diagnostic method or stroke classification. |
| Elderia 2023 | NA | NA | NA | NA |
| Alperi 2021 | Death from any cause during follow-up | Follow-up MI events classified as STEMI or NSTEMI type 1. Dates captured. | Defined as any new coronary revascularization (PCI or CABG). Causes included: in-stent restenosis, new lesion in treated or non-treated vessels. | Not defined. |
| Søndergaard 2019 | Defined as death from any cause during the follow-up period. | Defined as events adjudicated by a clinical events committee. No diagnostic thresholds or timing criteria were specified. | NA | Defined as all stroke events (fatal and non-fatal), with disabling stroke reported separately. Assessed by a neurologist and adjudicated per VARC-2 criteria. Classification into ischemic or hemorrhagic was not provided. |
| Baumbach 2019 | NA | NA | NA | NA |
| Barbanti 2018 | Defined as death from any cause during follow-up, based on Kaplan–Meier estimates and Cox regression up to 3 years. | Not defined. | NA | Defined as stroke events recorded at follow-up intervals. No information was provided regarding stroke type (fatal/non-fatal, ischemic/hemorrhagic) or diagnostic method. |
| Wendt 2013 | Defined as death from any cause during follow-up, ascertained via active follow-up with complete tracking (100% completeness). | NA | Defined as cardiac reintervention (e.g., valve or vascular procedures), but no detailed definition or procedural | NA |

MI, myocardial infarction; PCI, percutaneous coronary intervention; CABG, coronary artery bypass grafting; STEMI, ST-elevation myocardial infarction; NSTEMI, non–ST-elevation myocardial infarction; ICD-10-CM, International Classification of Diseases, 10th Revision, Clinical Modification; VARC-2, Valve Academic Research Consortium-2; NA, not available.

Long-term outcomes were defined as adverse events occurring during follow-up periods of ≥2 years after the index procedure. Definitions were extracted from each study’s methods, results, supplementary materials, or protocols. Clinical events were ascertained using inpatient and outpatient medical records, national healthcare databases (e.g., Medicare), or diagnostic coding systems (e.g., ICD-10-CM). In certain studies, outcome adjudication was conducted by an independent clinical events committee.

**Supplementary Table S5.** Newcastle-Ottawa Scale Quality Assessment for Included Cohort Studies.

| Study | Selection | | | | Comparability | | Outcome | | |  |
| --- | --- | --- | --- | --- | --- | --- | --- | --- | --- | --- |
|  | Representative-ness | Selection of  non-exposed | Ascertainment  of exposure | Outcome not present at start | Comparability on most important factors | Comparability on other risk factors | Assessment of outcome | Long enough follow-up (median≥1 year) | Adequacy  (completeness) of follow-up | Score |
| Taghiyev 2025 | * | * | * | * | * | * | * | * | * | 9 |
| Jagadeesan 2025 | * | * | * | * | * | * | * | * | * | 9 |
| Amat-Santos 2024 | * | * | * | * | * | * | * | * | * | 9 |
| Ullah 2023 | * | * | * | * | * | * | * | - | * | 8 |
| McInerney 2023 | * | * | * | * | * | * | * | - | * | 8 |
| Lérault 2023 | * | * | * | * | * | - | * | * | * | 8 |
| Elderia 2023 | * | * | * | * | * | * | * | - | * | 8 |
| Alperi 2021 | * | * | * | * | * | - | * | * | * | 8 |
| Baumbach 2019 | * | * | * | * | * | - | * | - | * | 7 |
| Barbanti 2018 | * | * | * | * | * | * | * | * | * | 9 |
| Wendt 2013 | * | * | * | * | * | - | * | * | * | 8 |
| *indicates criterion met; - indicates significant of criterion not met. | | | | | | | | | |  |

**Supplementary Table S6**. GRADE Assessment of Evidence Quality for Outcomes.

| **Outcome** | **No. of studies** | **RCT** | **Cohort** | **No. of participants** | **Risk of bias** | **Inconsistency** | **Indirectness** | **Imprecision** | **Publication bias** | **Plausible confounding** | **Magnitude of effect** | **Dose-response gradient** | **Quality** |
| --- | --- | --- | --- | --- | --- | --- | --- | --- | --- | --- | --- | --- | --- |
| 30-day All-cause mortality | 12 | 2 | 10 | 53,789 | no serious risk | serious inconsistency | no serious  indirectness | no serious  imprecision | undetected | would not reduce effect | no | no | Very low |
| 30-day Myocardial infarction | 6 | 2 | 4 | 3,344 | no serious risk | no serious inconsistency | no serious  indirectness | serious  imprecision | undetected | would not reduce effect | no | no | Very low |
| 30-day Stroke | 10 | 2 | 8 | 53,011 | no serious risk | no serious inconsistency | no serious  indirectness | serious  imprecision | undetected | would reduce effect | no | no | Low |
| 30-day Major vascular complications | 4 | 2 | 2 | 2,293 | no serious risk | serious inconsistency | no serious  indirectness | serious  imprecision | undetected | would not reduce effect | yes | no | Very low |
| 30-day Acute kidney injury | 4 | 1 | 3 | 39,943 | no serious risk | serious inconsistency | no serious  indirectness | no serious  imprecision | undetected | would not reduce effect | yes | no | Low |
| 30-day Major Bleeding | 4 | 2 | 2 | 12,106 | no serious risk | serious inconsistency | no serious  indirectness | serious  imprecision | undetected | would not reduce effect | no | no | Very low |
| 30-day Permanent pacemaker implantation | 7 | 2 | 5 | 51,719 | no serious risk | no serious inconsistency | no serious  indirectness | no serious  imprecision | undetected | would not reduce effect | yes | no | Moderate |
| All-cause mortality during follow-up | 7 | 1 | 6 | 39,422 | no serious risk | no serious inconsistency | no serious  indirectness | no serious  imprecision | undetected | would not reduce effect | no | no | Low |
| Myocardial infarction during follow-up | 5 | 1 | 4 | 39,179 | no serious risk | serious inconsistency | no serious  indirectness | no serious  imprecision | detected | would reduce effect | yes | no | Low |
| Stroke during follow-up | 4 | 1 | 3 | 38,707 | no serious risk | no serious inconsistency | no serious  indirectness | no serious  imprecision | undetected | would not reduce effect | no | no | Low |
| Revascularization during follow-up | 4 | 0 | 4 | 38,849 | no serious risk | no serious inconsistency | no serious  indirectness | no serious  imprecision | undetected | would not reduce effect | yes | no | Moderate |


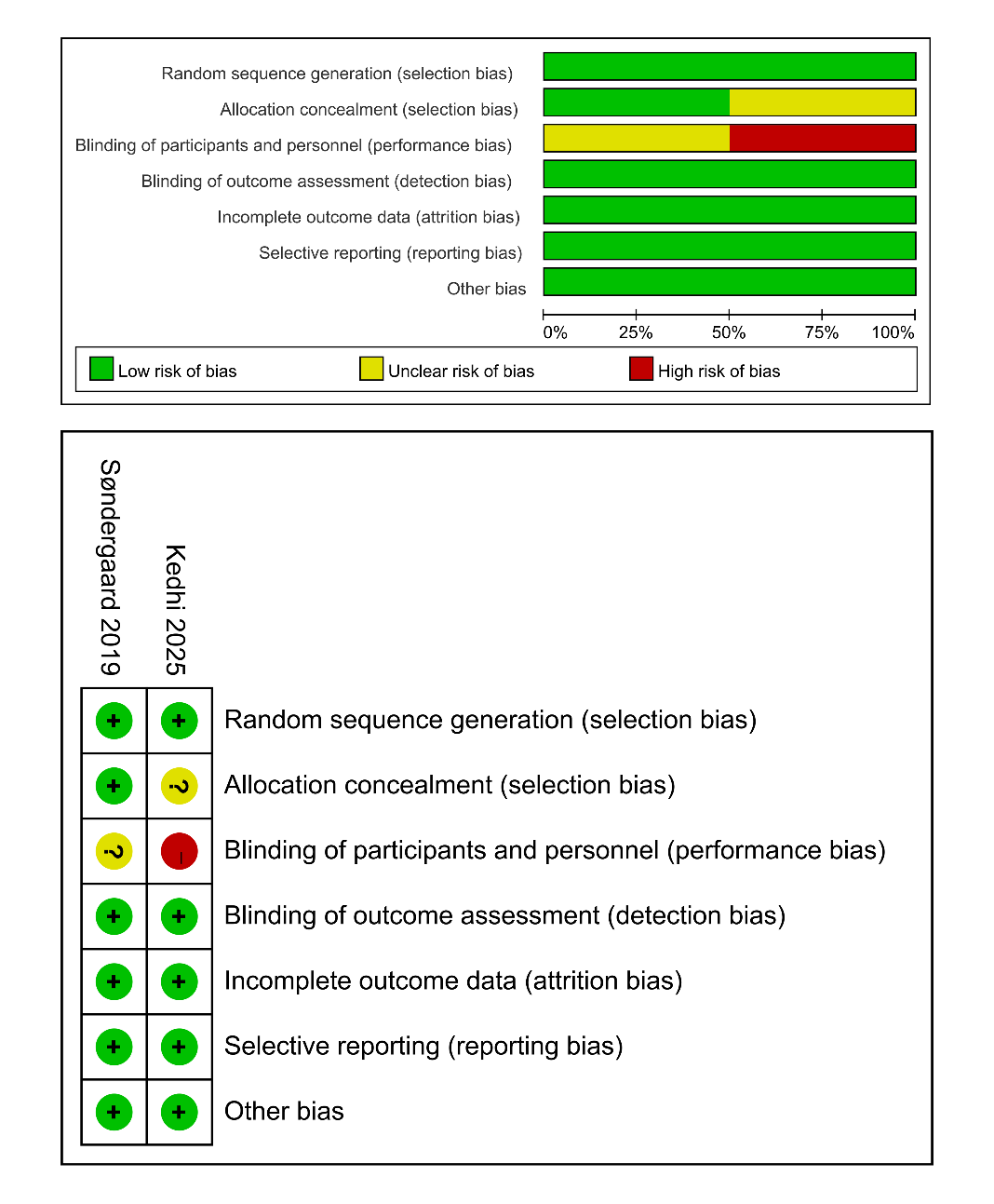


**Supplementary Figure S1.** Risk of Bias Assessment for RCTs.

**
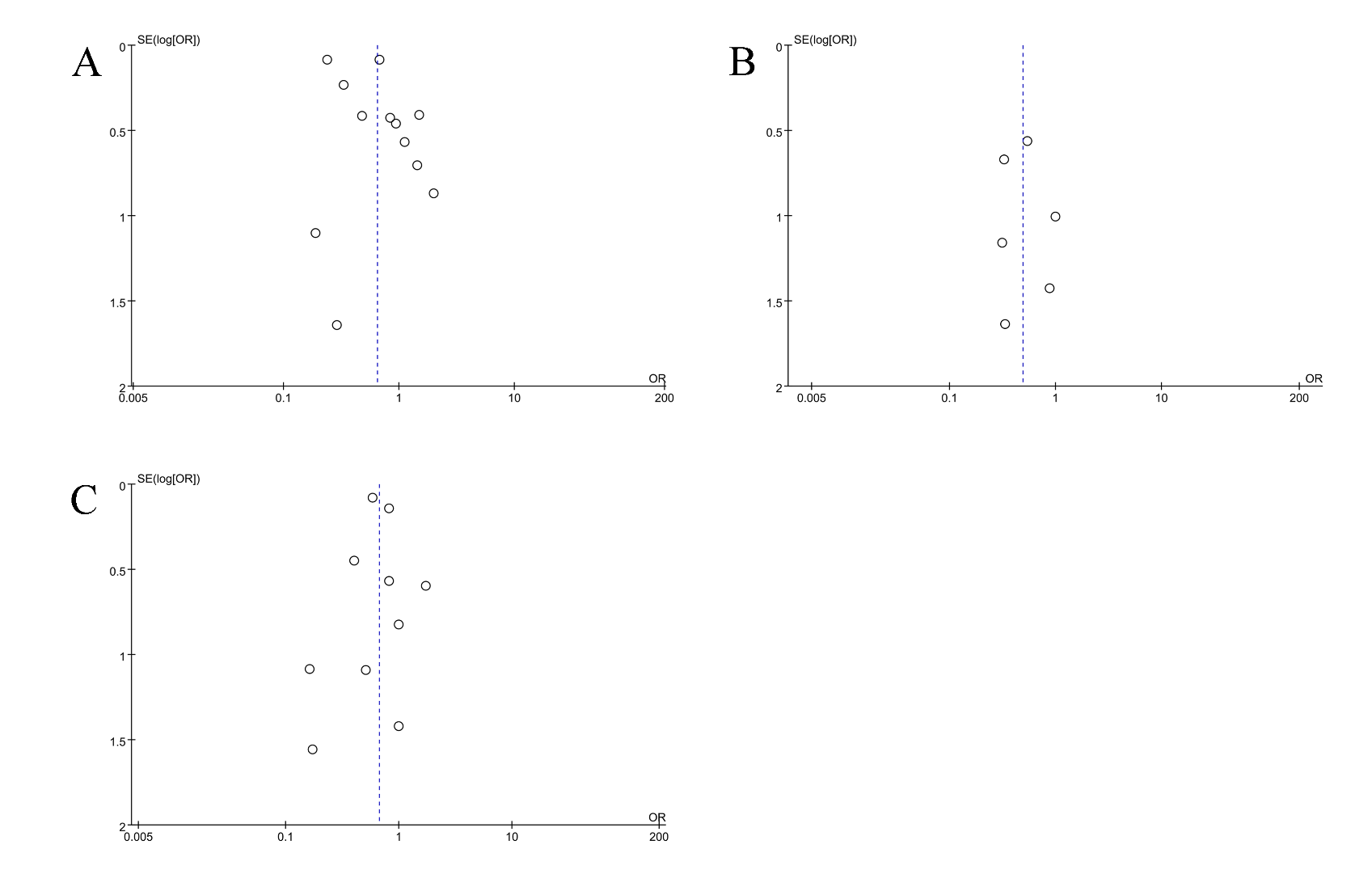
**

**Supplementary Figure S2.** Funnel Plots for Publication Bias in Short-Term Outcomes in Intermediate- and High-Risk Patients Undergoing TAVR+PCI vs. SAVR+CABG.

(A) 30-day all-cause mortality, (B) 30-day myocardial infarction, and (C) 30-day stroke.

**
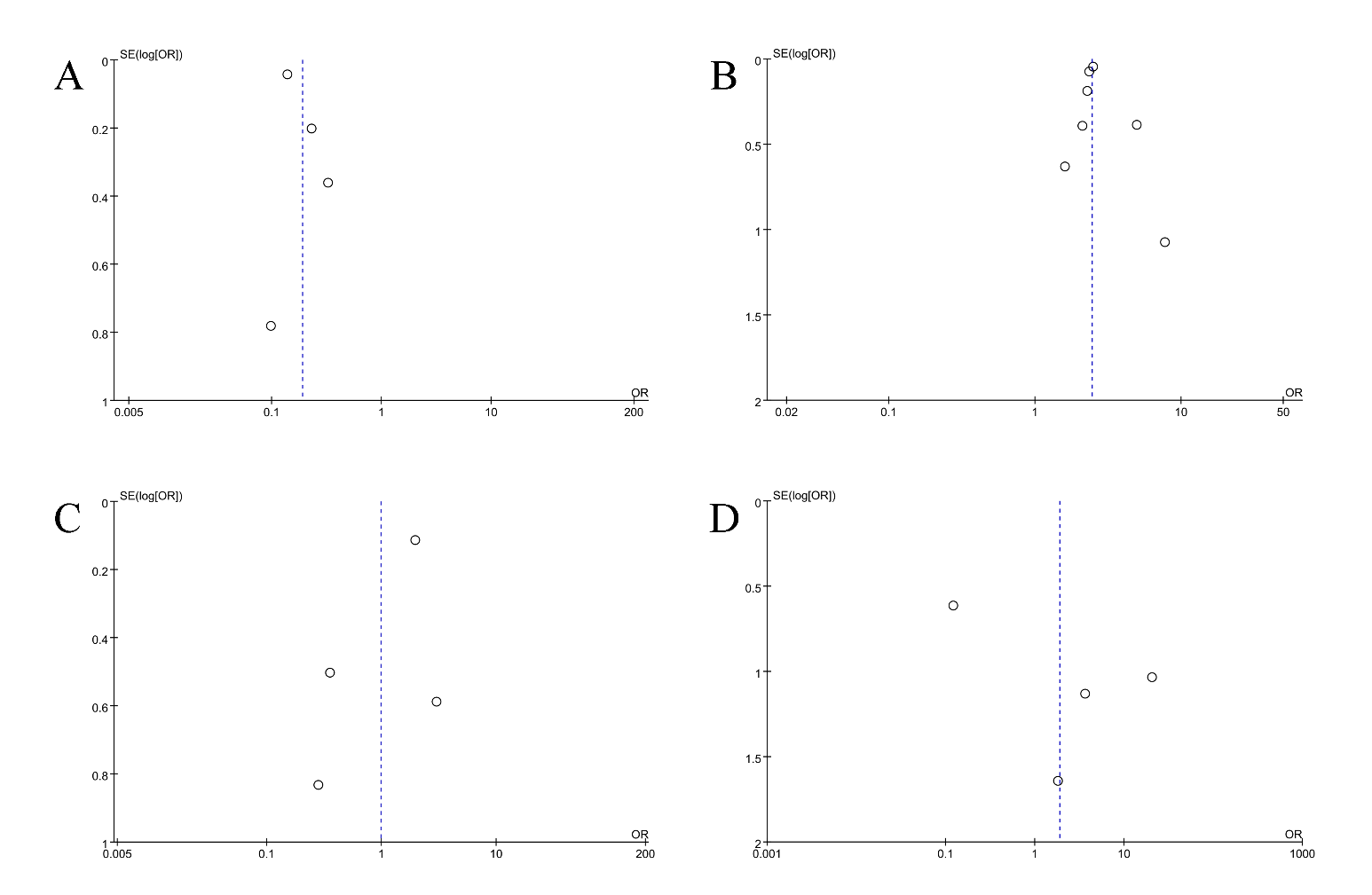
**

**Supplementary Figure S3.** Funnel Plots for Publication Bias in Short-Term Procedural Complications in Intermediate- and High-Risk Patients Undergoing TAVR+PCI vs. SAVR+CABG.

(A) 30-day acute kidney injury, (B) 30-day permanent pacemaker implantation, (C) 30-day major bleeding, and (D) 30-day major vascular complications.

**
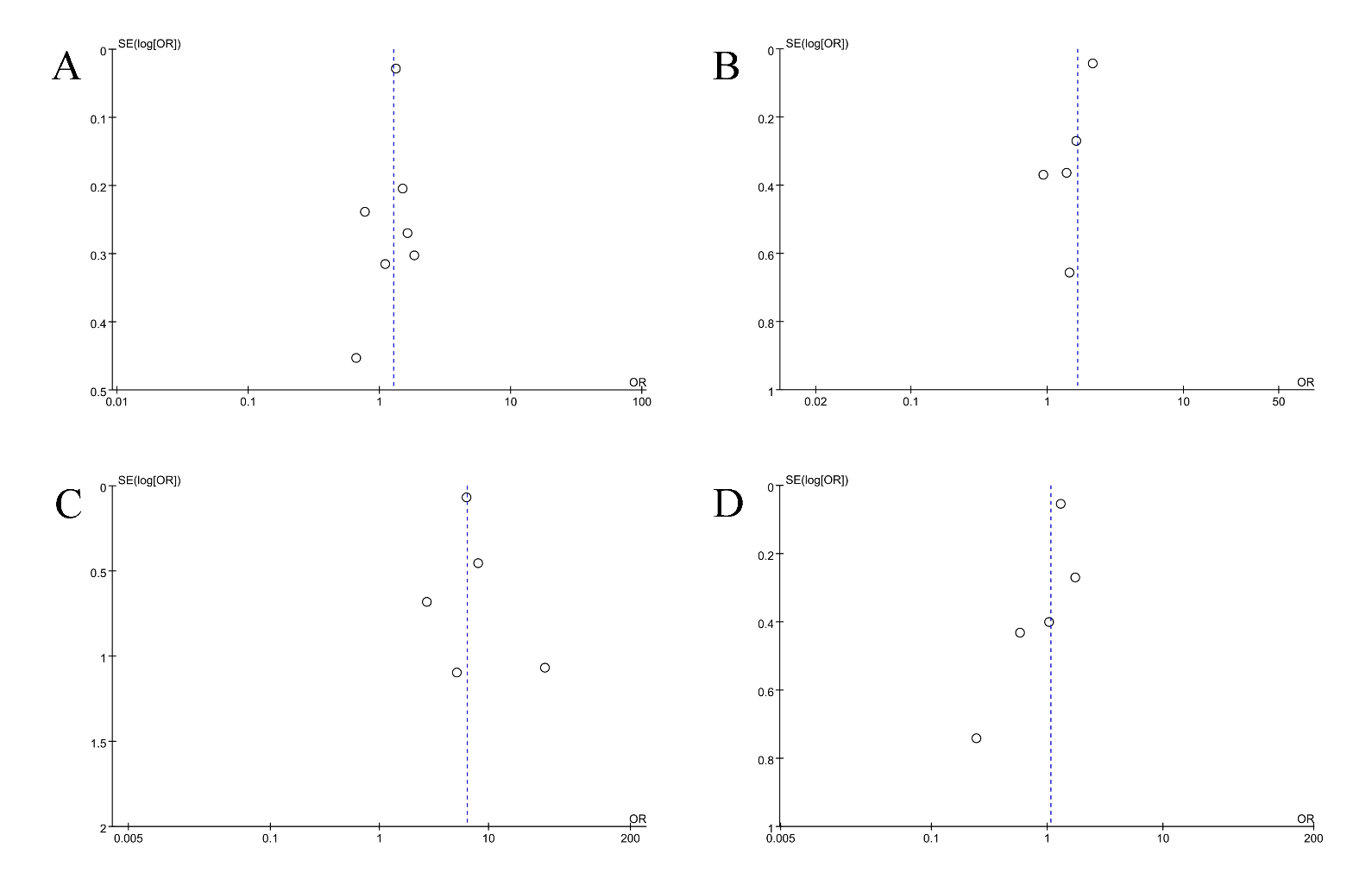
**

**Supplementary Figure S4.** Funnel Plots for Publication Bias in Mid- to Long-Term Outcomes in Intermediate- and High-Risk Patients Undergoing TAVR+PCI vs. SAVR+CABG.

(A) All-cause mortality during follow-up, (B) Myocardial infarction during follow-up, (C) Revascularization during follow-up, and (D) Stroke during follow-up.


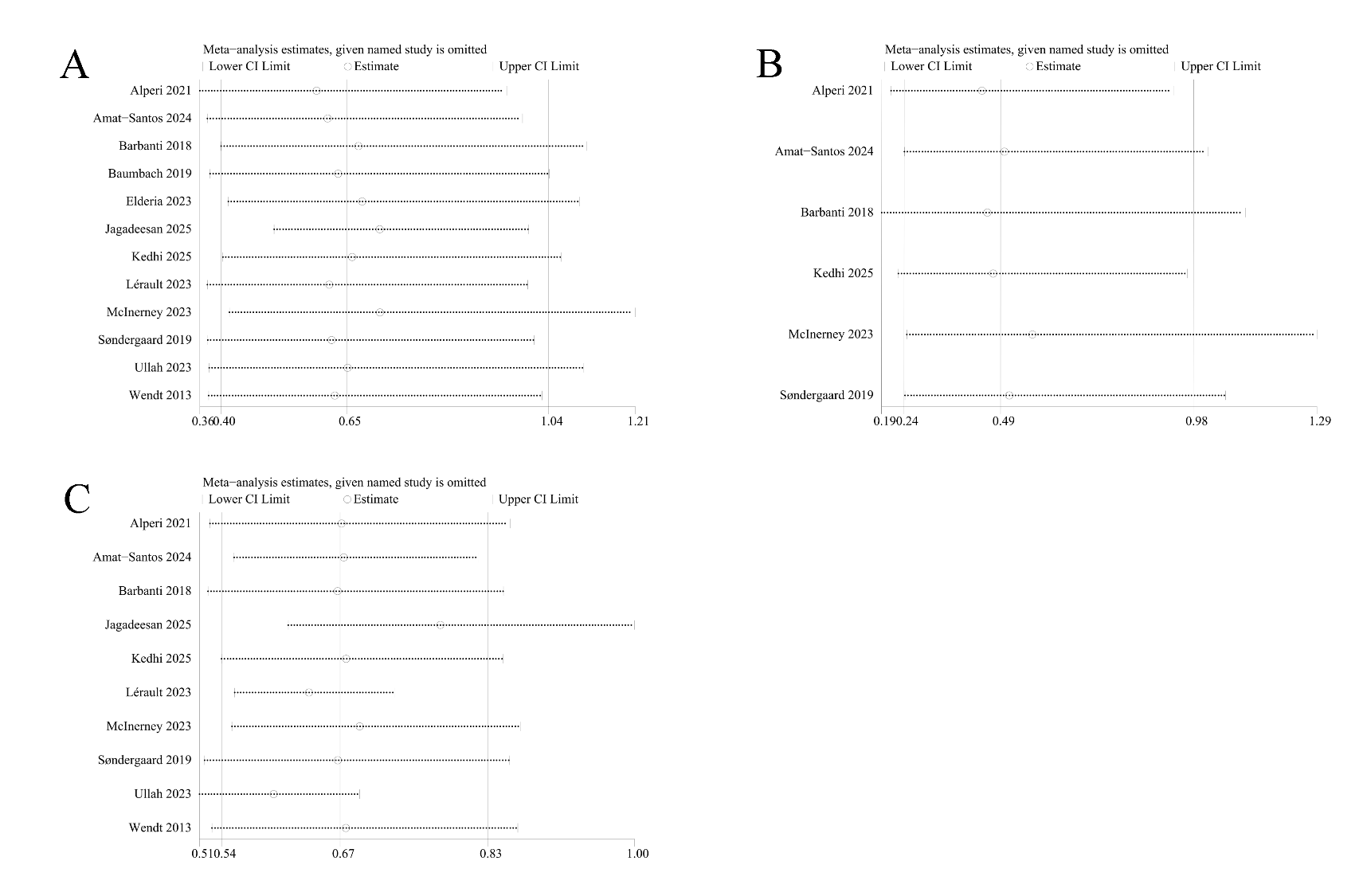


**Supplementary Figure S5.** Sensitivity Analysis for Short-Term Outcomes in Intermediate- and High-Risk Patients Undergoing TAVR+PCI vs. SAVR+CABG.

(A) 30-day all-cause mortality, (B) 30-day myocardial infarction, and (C) 30-day stroke.

**
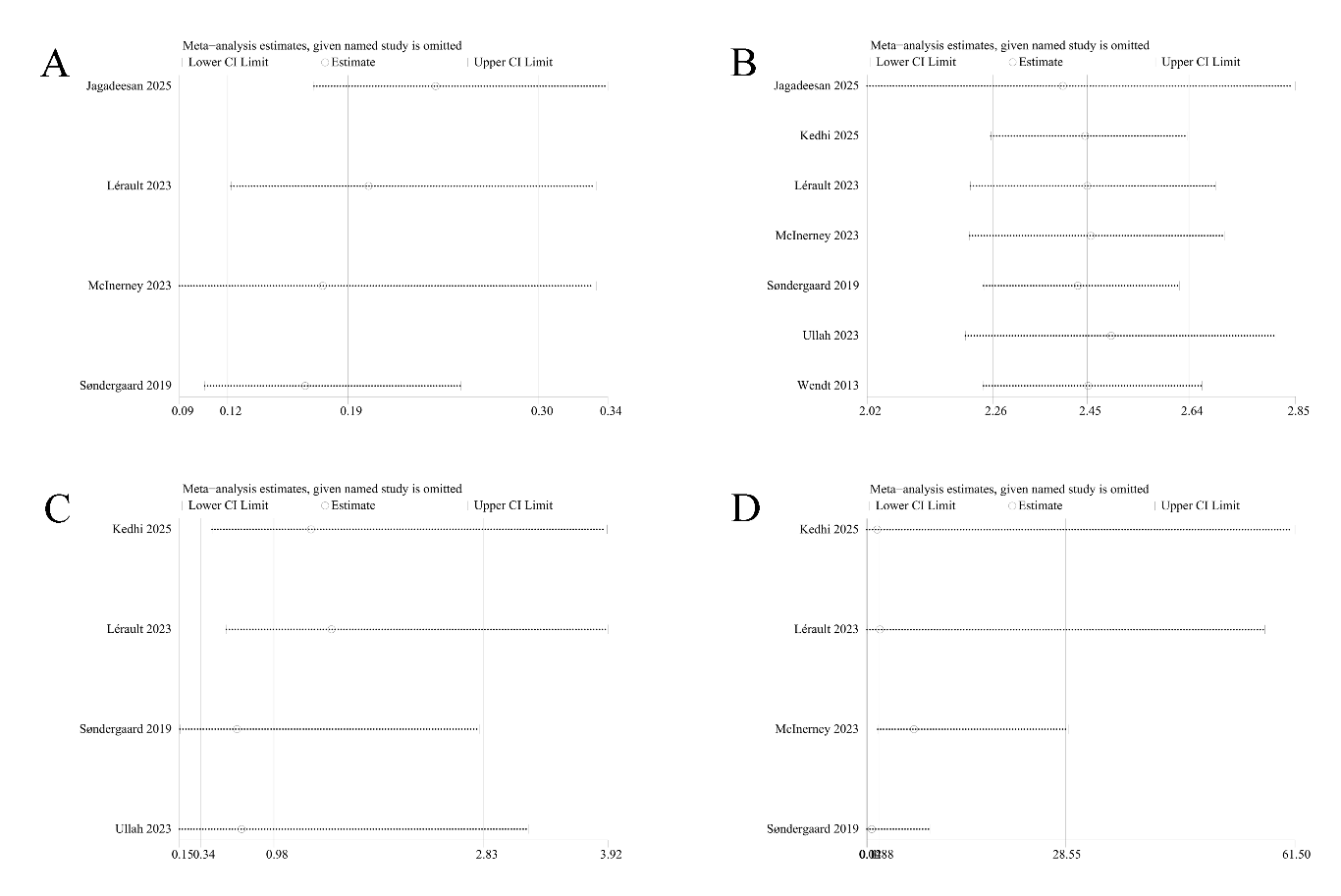
**

**Supplementary Figure S6.** Sensitivity Analysis for Short-Term Procedural Complications in Intermediate- and High-Risk Patients Undergoing TAVR+PCI vs. SAVR+CABG.

(A) 30-day acute kidney injury, (B) 30-day permanent pacemaker implantation, (C) 30-day major bleeding, and (D) 30-day major vascular complications.

**
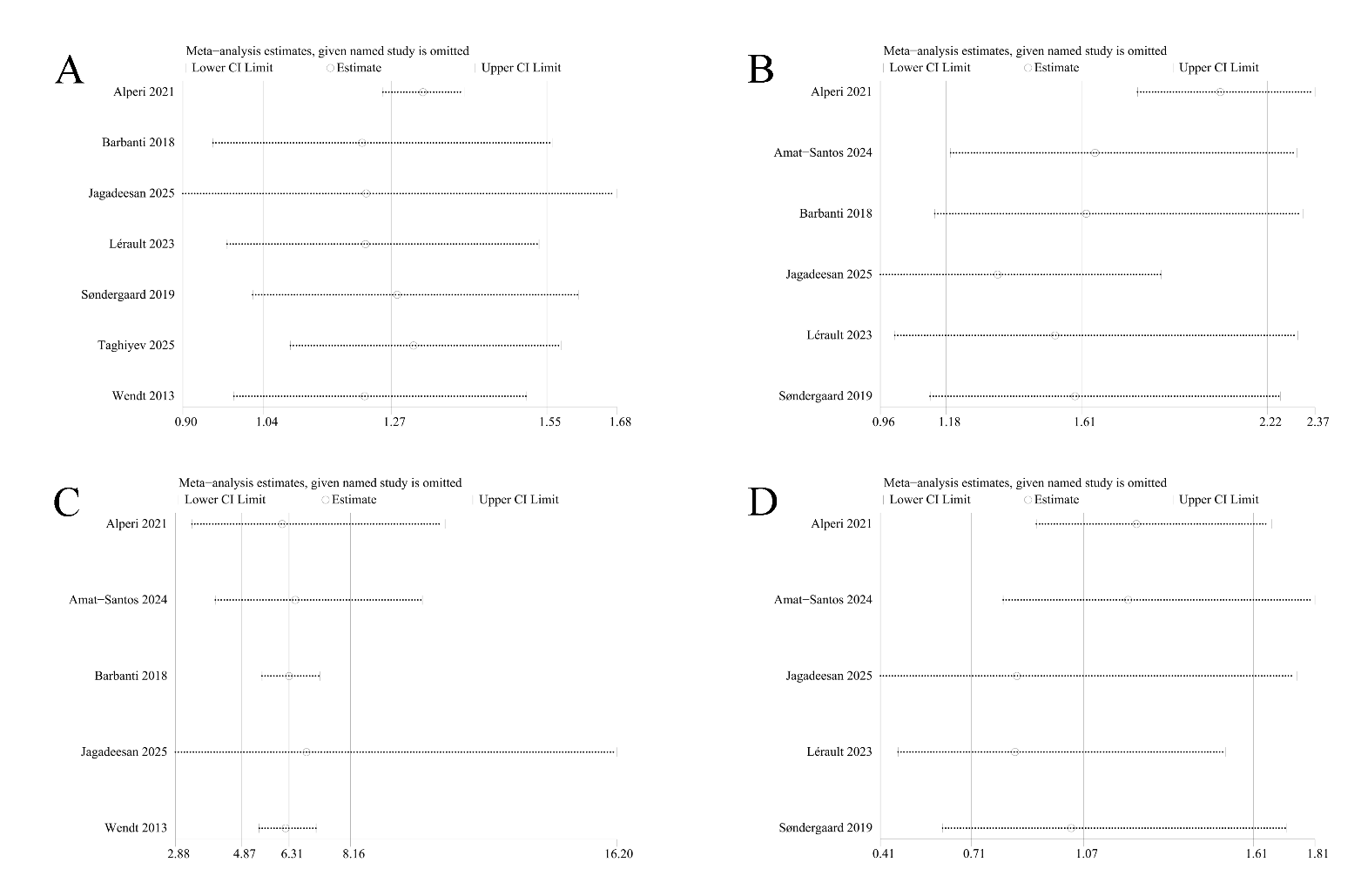
**

**Supplementary Figure S7.** Sensitivity Analysis for Mid- to Long-Term Outcomes in Intermediate- and High-Risk Patients Undergoing TAVR+PCI vs. SAVR+CABG.

 (A) All-cause mortality during follow-up, (B) Myocardial infarction during follow-up, (C) Revascularization during follow-up, and (D) Stroke during follow-up.
